# Symptomatology and IgG Levels before and after SARS-CoV-2 Omicron Breakthrough Infections in Vaccinated Individuals

**DOI:** 10.3390/vaccines12101149

**Published:** 2024-10-08

**Authors:** Nigella M. Paula, Emerson Joucoski, Valter A. Baura, Emanuel M. Souza, Fabio O. Pedrosa, Alan G. Gonçalves, Luciano F. Huergo

**Affiliations:** 1 Setor Litoral, Federal University of Paraná—UFPR, Matinhos 83260-00, PR, Brazil; nigellamenp@hotmail.com (N.M.P.); joucoski@gmail.com (E.J.); alan.goncalves@ufpr.br (A.G.G.); 2 Graduated Program in Sciences-Biochemistry, Federal University of Paraná—UFPR, Curitiba 81530-00, PR, Brazil; baura@ufpr.br (V.A.B.); souzaem@ufpr.br (E.M.S.); fpedrosa@ufpr.br (F.O.P.); 3 Graduated Program in Farmacy-Biochemistry, Federal University of Paraná—UFPR, Curitiba 81530-00, PR, Brazil

**Keywords:** omicron, symptoms, IgG, SARS-CoV-2, COVID-19

## Abstract

(1) Background: After the COVID-19 pandemic, there is concern regarding the immunity of the population to SARS-CoV-2 variants, particularly the Omicron variant and its sub-lineages. (2) Methods: The study involved analyzing the immune response and symptomatology of 27 vaccinated individuals who were subsequently infected by Omicron sub-lineages. Blood samples were collected for serological analysis, including the detection of IgG antibodies reactive to the Nucleocapsid (N) and Spike (S) antigens of SARS-CoV-2. Additionally, participants were interviewed to assess the intensity of symptoms during the infection. (3) Results: Despite the high levels of anti-Spike IgG observed after vaccination, all participants were infected by Omicron sub-lineages. The most common symptoms reported by participants were fever or chills, sore throat, and cough. The levels of anti-Spike IgG found prior to infection did not correlate with symptom intensity post-infection. However, it was observed that high post-infection anti-Nucleocapsid IgG levels correlated with mild symptoms during the course of the disease, suggesting a potential role for anti-N antibodies in symptom intensity. (4) Conclusions: In line with previous studies, the high levels of IgG anti-Spike resulting from vaccination did not provide complete protection against infection by the Omicron variant. Additionally, our data suggest that anti-Nucleocapsid IgG titers are negatively correlated with the intensity of the symptoms during mild infections.

## 1. Introduction

After more than three years since the onset of the COVID-19 pandemic, attention has turned to the immunity of the population, either elicited by previous infections and/or by vaccination in light of emerging SARS-CoV-2 virus variants. Observational studies and clinical trials confirm the remarkable effectiveness of SARS-CoV-2 vaccines in preventing severe COVID-19 symptoms [1,2,3,4,5,6,7]. Quite remarkably, the levels of IgG antibody against the SARS-CoV-2 Spike protein seem to correlate with protection against symptomatic or severe COVID-19 [8,9,10].

In our previous studies, we demonstrated the excellent performance of the Brazilian COVID-19 vaccination program in inducing humoral immunity as measured by the levels of IgG reactive to SARS-CoV-2 Spike protein. We also reported a higher performance of the BNT162b2 vaccine (Pfizer-BioNTech) in inducing anti-Spike IgG antibodies after the initial vaccination and booster dose in comparison to other vaccines such as ChadoX and Coronavac [11], findings that have been confirmed by other studies [12,13,14].

It is important to note that the levels of IgG reactive to the SARS-CoV-2 Spike protein tend to decrease after vaccination, which may result in reduction in protection against novel SARS-CoV-2 infections and symptomatic COVID-19 [15,16,17,18]. Similarly, although prior SARS-CoV-2 infection provides protection against reinfection [19,20,21], such protection also gradually diminishes [15,22].

SARS-CoV-2 Variants of Concern (VOCs), especially those carrying amino acid substitutions in the Spike protein, as observed in Omicron variants (B.1.1.529/BA, and sub-lineages BA.1, BA.2, BA.4, and BA.5), have the potential to overcome adaptive immunity elicited against the original SARS-CoV-2 epitopes [23,24]. Decreased vaccine-based protection has been reported for the Omicron variants, which became dominant on a global scale [25,26]. In Brazil, since the beginning of 2022, the Omicron variant and its sub-lineages have led to a significant increase in infection rates among fully vaccinated as well as boosted individuals due to their high transmission capacity and potential for immune escape [27,28,29].

Infection by Omicron variants usually results in asymptomatic and/or mildly symptomatic infections. The attenuation in the severity of symptoms observed in previously vaccinated individuals, compared to those not vaccinated, is presumably attributed to the partial protection provided by the residual repertoire of neutralizing antibodies (nAb) and the activation of the memory of B and T cells acquired during the vaccination process [30,31]. Omicron variants may cause less severe infections due to pre-mounted memory immunity and the predisposition of these variants to primarily infect the upper respiratory tract [32,33]. Subtle clinical symptoms caused by Omicron variants often result in underdiagnosis, contributing to the continued global spread and evolution of SARS-CoV-2 [34,35].

Despite comprehensive research on the impact of SARS-CoV-2 viral mutations and their transmission capability [36,37,38], more data are needed to understand how the previous humoral response, mounted against the original SARS-CoV-2 virus, correlates with infection and symptomatology to emerging variants, such as Omicron.

Here we analyzed the symptomatology and IgG levels before and after SARS-CoV-2 Omicron breakthrough infections in vaccinated individuals to understand the impact of previous immunity on the symptomatology of Omicron sub-lineage infections.

## 2. Materials and Methods

### 2.1. Study Design

This is a case series on SARS-CoV-2 breakthrough infections, selected from a previous cohort a “population analysis of” COVID-19 IgG levels [11,39]. Sampling campaigns took place between 2021 and 2022, at the Federal University of Paraná, Matinhos, Brazil. Individuals aged 18 and over were invited to participate in the study, with the flexibility to choose when and with what frequency they would volunteer for the sampling campaigns. Enrollments were made through the study’s website. Participants provided their consent, along with a range of personal information including: previous SARS-CoV-2 infections before enrolment during the study (if any); a positive SARS-CoV-2 test during this study (if any, diagnosed by an official qRT-PCR and/or antigen test); details of the vaccination schedule (including the vaccine manufacturer) and the dates of administered doses.

From a cohort of 1785 individuals in the current study, we selected 27 individuals who participated in at least three different sampling campaigns and underwent primary vaccination (first and second doses) and at least one booster dose (third dose). The selected participants contracted SARS-CoV-2 infection naturally after the primary vaccination and/or booster doses.

The participants were asked to fill out an investigation form indicating the presence of underlying health conditions, symptoms, and their intensity if present (Table S1). Symptom intensity was assessed using a designed a scale from zero to 10, reflecting each participant’s individual perception, where zero indicated the absence of symptoms and 10 indicated very intense symptoms. This approach allowed for a subjective evaluation tailored to each participant’s perception of symptom intensity. The symptomatology described was based on the latest update from the Brazilian Ministry of Health (https://www.gov.br/saude/pt-br/assuntos/covid-19/sintomas—Accessed on 15 August 2024).

### 2.2. Serological Analysis

As detailed in our previous studies [40,41], serological analysis involved the collection of blood samples through capillary puncture. The samples were centrifuged, and four microliters of serum were used to investigate IgG antibodies reactive to the Nucleocapsid (anti-N) and Spike (anti-S) antigens of SARS-CoV-2. For the analysis, 1 mg of His-tagged antigens was incubated with 1 mL of nickel magnetic particles (Promega-V8565) in 50 mL of TBST for 5 min at room temperature with gentle mixing. The beads were washed with 25 mL of TBST and resuspended in 5 mL of TBST.

The magnetic bead immunoassay was conducted using a 96-sample format with flat bottom plates. The 0.8 mL aliquots of antigen-loaded beads were resuspended in 11 mL of TBST containing 1% (*w*/*v*) skimmed milk, and 0.1 mL of this mixture was distributed into each well of a 96-well plate. Four microliters of human serum were diluted in 0.2 mL of TBST with 1% (*w*/*v*) skimmed milk directly in the wells of another 96-well plate. The magnetic beads were transferred to the sample plate and incubated with the human serum for 2 min with gentle mixing. The beads were captured and subjected to two sequential wash steps for 30 s in TBST. They were then incubated for 2 min with 0.15 mL of goat anti-human IgG-PE, diluted 1:250 in TBST, followed by two washing steps of 30 s each in TBST. Finally, the beads were transferred to a plate containing 0.15 mL of TBST per well, homogenized for 10 s, and analyzed using a TECAN M Nano plate reader with excitation at 545 nm and emission at 578 nm. The IgG titers were reported as normalized values as percentage of a reference positive serum. The detecting antibodies against Spike and Nucleocapsid operated with 96.8% sensitivity (95% CI 89.0–99.6) and 99.5% specificity (95% CI, 97.7–99.9%) [41]. The method was highly correlated with data obtained using the MagPlex system (see reference [42] for details) and outperformed classic ELISA [41,43].

The occurrence time of SARS-CoV-2 infections in volunteers was informed by the time of an official positive RT-qPCR or antigen test, if available. We considered that “possible SARS-CoV-2 infection” had occurred on those individuals who seroconverted to IgG reactive to Nucleocapsid during this study. Individuals who seroconverted to anti-N IgG after receiving a shot of Coronavac were excluded as this vaccine can result in Nucleocapsid seroconversion [44]. Although it is not possible to determine the precise date of these possible infections, they were reported to occur 10 days before the detected anti-N IgG seroconversion. Vaccination of volunteers was reported on graphics according to the date of each dose and respective manufacturer as reported by each participant.

### 2.3. Variant Identification

For most of the individuals, the SARS-CoV-2 variant that caused the infection was presumed based on the month of the reported infection, along with the data indicating the dominant variant that circulated in the state of Paraná, Brazil, during the infection event. The data were retrieved from the Fiocruz Genomic Network—GISAID (https://www.genomahcov.fiocruz.br/—Accessed in October 2023) and Nextstrain (https://nextstrain.org/ncov/gisaid/south-america/all-time?dmax=2022-11-07&dmin=2022-01-01&f_country=Brazil&l=clock&regression=hide—Accessed in October 2023).

A sample from one individual, as detailed in the text, was subject to SARS-CoV-2 genome sequencing using the Illumina platform. In brief, viral RNA was extracted from 0.2 mL of saliva samples using an automated magnetic extractor and the MagMAX™ Pathogen RNA/DNA Kit (Applied Biosystems™, Waltham, MA, USA). Initial confirmation of SARS-CoV-2 in the sample was performed by RT-qPCR using Kit Biomol OneStep/COVID-19 PCR Master Mix Reverse Transcription Kit (IBMP) on a QuantStudio5 instrument (Thermo Fisher Scientific™ Waltham, MA, USA). After confirming the presence of SARS-CoV-2 by RT-qPCR, the extracted RNA was subjected to cDNA synthesis and amplification of the viral genome by PCR in overlapping fragments of approximately 300 base pairs, with two primer pools provided in the Illumina COVIDSeq Test kit (Illumina, San Diego, CA, USA). The viral genome was sequenced using reagents from the Illumina COVIDSeq Test kit and Nextseq 1000/2000 instrument (Illumina, San Diego, USA).

The raw data were processed using the COVID Lineage pipeline on the Dragen analysis platform. After quality control, the consensus sequence was assembly and mapped against the reference strain (NC_045512.2) to determine the identity of the mutations. The classification of lineages followed the dynamic classification system proposed by Rambaut and collaborators (2020) [45], through the Phylogenetic Assignment of Named Global Outbreak LINages software, available at (https://github.com/cov-lineages/pangolin, accessed on 10 August 2024) and also through NextClade. Definitive classification of lineages was made after confirmation by phylogenetic analysis containing representative sequences of the main circulating lineages.

### 2.4. Data Analysis

The statistical analyses were conducted using the software NCSS 11 and GraphPad Prism (version 8.4.3). Initially, we used descriptive statistics to understand the data characteristics. Subsequently, we employed Spearman correlation analysis to explore relationships between variables. For group comparisons, we utilized Tukey’s ANOVA test. The Kruskal–Wallis ANOVA test was employed when assumptions of normality or equal variance were not met.

## 3. Results

### 3.1. Cohort Description

Among the 1785 participants from our previously populational studies we identified 27 participants who had been subjected to at least three rounds of serological analysis, had been fully vaccinated, and had a SARS-CoV-2 infection after completing the primary and/or booster vaccination. This group had an average age of 40 years (SD 12; ranging from 19 to 74 years) with 70% being women (Table 1). All participants completed the primary vaccination for COVID-19 during the study; 81% had taken the third and 19% up to the fourth booster dose. Vaccination schemes covered both homologous and heterologous combinations from different manufacturers (Table 1).

Among the selected 27 individuals, five mentioned having a previous SARS-CoV-2 infection before enrollment in this study. All the cohort (*n* = 27) underwent a SARS-CoV-2 infection during this study; 59% of the cases were diagnosed by an official qRT-PCR and/or antigen test and were symptomatic (Table 1). The remaining 41% of the individuals underwent asymptomatic SARS-CoV-2 infection which was tracked during this study by anti-N IgG seroconversion. All, except one participant (code B), seroconverted to anti-S IgG before the SARS-CoV-2 infection event. Relevant information from all individuals is provided in Table 1, with more details in Table S2.

### 3.2. Time Course IgG Titers, Variant and Disease Symptomatology

**Figure 1:**
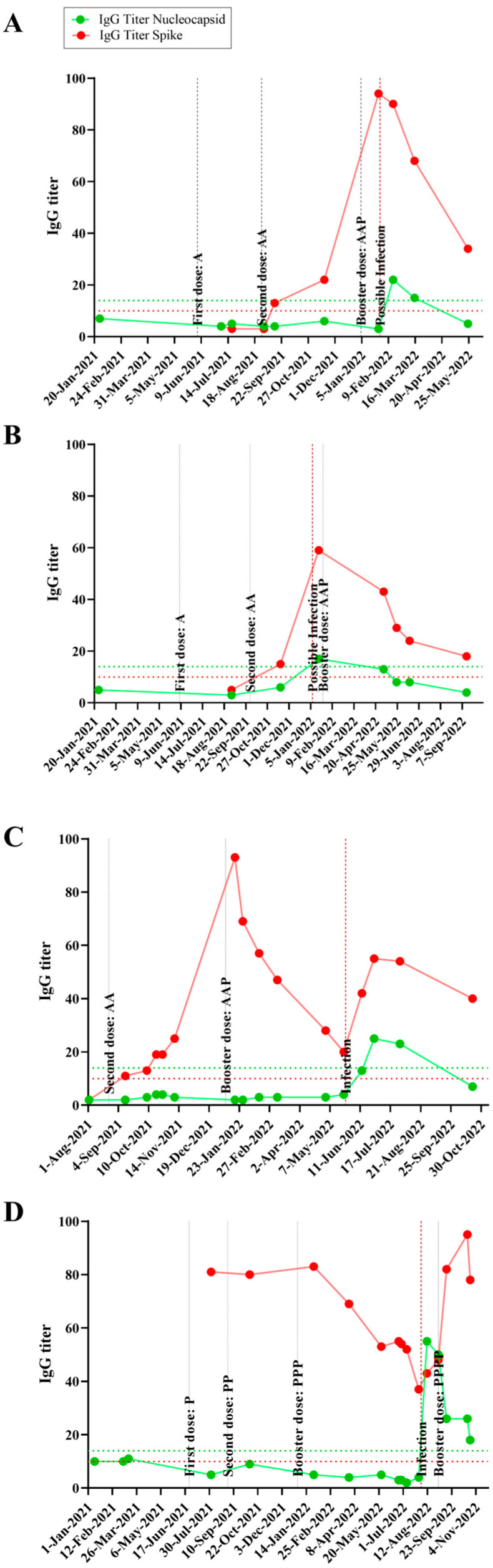
Evolution of IgG levels before and after SARS-CoV-2 breakthrough infections in vaccinated individuals. The *x*-axis indicates the temporal analysis of each individual; infection and vaccination events are indicated by vertical lines. Each vaccine dose and the vaccination scheme are reported according to the initial letter of the vaccine’s name (A, for AstraZeneca, C for CoronaVac, J for Janssen, P for Pfizer). The anti-N (green) and anti-S (red) IgG titers are shown on the left *y*-axis, dashed lines indicate the positivity cutoff. (**A**,**B**) asymptomatic cases; (**C**,**D**) symptomatic cases.

The time course, IgG titers and relevant events occurring in each individual are graphically expressed in Figure S1. Specific examples, focusing on four individuals, are shown in Figure 1, two of which were asymptomatic (panels A and B) and two were symptomatic for SARS-CoV-2 infection (panels C and D). The x-axis shows the temporal analysis of each individual with infection and vaccination events indicated by vertical lines. The anti-N and anti-S IgG titers followed the expected pattern, with anti-S titers raised after a vaccination event, whereas both anti-S and anti-N levels increased after an infection event. In the absence of further infection and/or vaccination both anti-S and anti-N titers waned over time, with anti-N showing a steeper decrease so that it became undetectable a few months after infection in most individuals (Figure 1 and Figure S1).

Quite remarkably, the data depicted in Table 1 and Table S2, Figure 1 and Figure S1 show that SARS-CoV-2 infections can occur even in individuals who had high anti-S IgG titers before the infection. During the course of the present study, all the vaccines in use in Brazil were produced based on the original Wuhan SARS-CoV-2 strain. Several studies showed that emerging SARS-CoV-2 variants, particularly Omicron, could escape the immune response which was elicited against the original version of the virus, considering either natural infections and/or vaccination.

To determine which SARS-CoV-2 variant was causing the infections, the date of each infection event was correlated with the predominant SARS-CoV-2 strain, with the predominancy data obtained from the Fiocruz Genomic surveillance Network–GISAID (https://www.genomahcov.fiocruz.br/—Accessed in 10 October 2023) and Nextstrain (https://nextstrain.org/ncov/gisaid/south-america/all-time?dmax=2022-11-07&dmin=2022-01-01&f_country=Brazil&l=clock&regression=hide—Accessed in 10 October 2023) (Figure S2). This analysis indicates that the Omicron variant caused all the cases reported in this study as this strain was far dominant during this study (Figure S2). The estimated frequencies of 10%, 40% and 50% for the BA.1, BA.2 and BA.5 variants, respectively (Table 1 and Table S2).

A saliva sample collected during the active infection of one individual (Table S2, code N) was subjected to SARS-CoV-2 genome sequencing which identified BA.2 (supplementary information) as the infecting variant, corroborating the prediction based on strain predominance over time (Table 1 and Table S2). The genome sequence-based strain identification can also apply to individuals L, P and Y (Table 1 and Table S2), since these cases were related to individual N (Figure S3).

**Figure 2:**
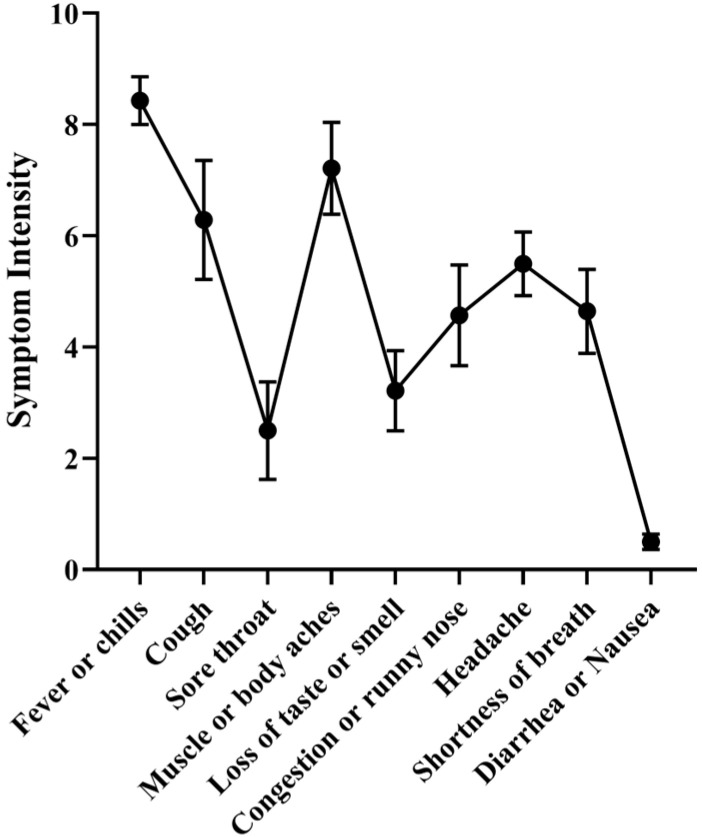
Intensity of each of the COVID-19 symptoms reported by the participants on a scale from 0 to 10. Data show mean values, and SEM (standard error of the mean).

Given that the cohort of this study had completed the vaccination scheme before being infected with different SARS-CoV-2 omicron variants, it would be interesting to understand the symptom intensity of the infection event. All the symptomatic participants answered a form where they rated nine different COVID-19 related symptoms (Table S1) on a scale from zero (no symptoms) to 10 (most severe symptom). The three most common symptoms were: fever or chills; sore throat; cough, with an average intensity of 7.9, 6.8 and 6.7, respectively (Figure 2 and Table S3). The sum of the intensity of all the symptoms was used as a proxy for the symptom intensity of each infection. The average was 42 (SD 13; ranging from 22 to 69).

### 3.3. Correlation between IgG Levels and COVID-19 Symptomatology

Several studies indicate that anti-S IgG titers elicited in response to vaccination and/or to previous infection correlate with protection against SARS-CoV-2 infections and symptoms. In this context, it is important to ascertain whether the IgG elicited against the Spike antigen of the original Wuhan isolate can provide protection against infections by emerging variants of concern, including Omicron.

**Figure 3:**
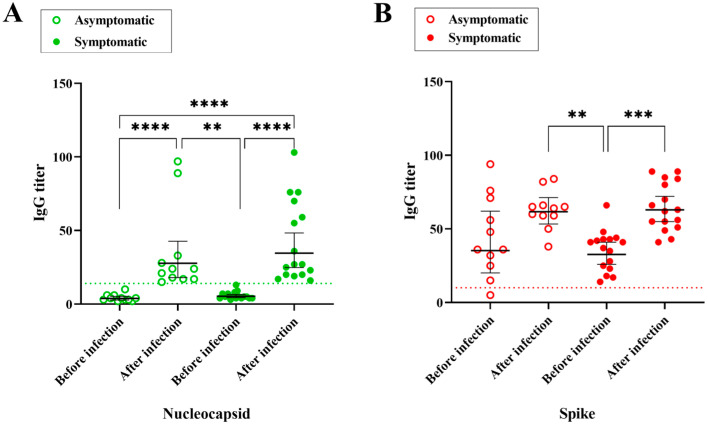
Correlation between (**A**) IgG Nucleocapsid (anti-N) and (**B**) Spike (anti-S) titers before and after the last COVID-19 infection, comparing asymptomatic and symptomatic groups. The bars represent the geometric mean with 95% CI; the dashed line indicates the seropositivity cutoff point. One-way ANOVA analysis was performed and significant values are indicated **** (*p* < 0.0001); *** (*p* = 0.0005) and ** (*p* < 0.0034).

Within the cohort of this study, all except one participant (code B, Table S2) seroconverted to anti-S IgG as a consequence of vaccination prior to SARS-CoV-2 infection (Figure 3). The average anti-S IgG titer was 39.2 (SD 20), much higher than the assay cut-off which was 10. All 27 individuals were negative for anti-N IgG before infection, with an average anti-N signal of 5.1 (SD 2.5). As expected, all individuals seroconverted to anti-N IgG after infection, with an average anti-N signal of 39 (SD 28) and showed higher average anti-S IgG titers, with an average signal of 64 (SD 15) (Figure 3).

Quite remarkably, anti-S IgG titers before the infection were similar among the symptomatic and asymptomatic groups (*p* ≥ 0.99), with average anti-S IgG titers of 35 and 45, respectively. We noted that even participants having high pre-infection anti-S IgG titers underwent symptomatic infections (See Figure S1), thereby confirming SARS-CoV-2 Omicron variants were able to escape the humoral response elicited against the previous version of the virus.

**Figure 4:**
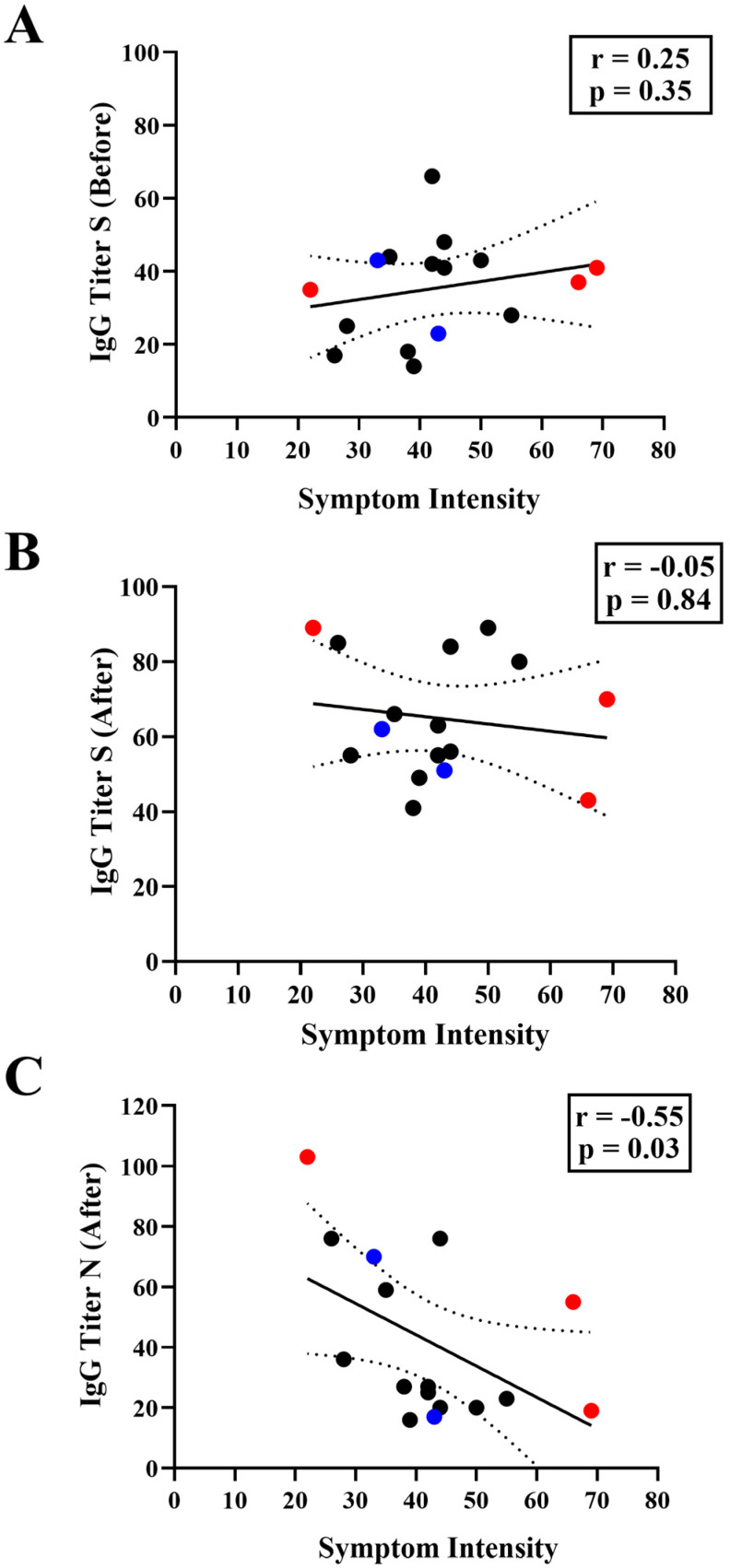
Correlation between IgG titers and symptom intensity. The symptom intensity (x-axis) is plotted against the IgG titers (y-axis) reactive to the Spike antigen (anti-S) observed before (**A**) and after (**B**) a SARS-CoV-2 infection event. (**C**) Correlation between the symptom intensity (x-axis) vs the IgG titers (y-axis) reactive to the Nucleocapsid antigen (anti-N) observed after a SARS-CoV-2 infection event. The symptom intensity of the infection was determined by the sum of frequencies related to the self-reported intensity level (scale of 0 to 10) of each symptom described by the participants of this study (Table S1). Blue dots indicate individuals who had SARS-CoV-2 infection after primary vaccination. Red dots indicate individuals with hybrid immunity (individuals reporting a previous SARS-CoV-2 infection before enrollment in this study) and who had SARS-CoV-2 infection after booster vaccination Black dots indicate individuals who had SARS-CoV-2 infection after a booster dose. The graph indicates the Spearman correlation coefficient with 95% CI.

We sought to determine if there was a negative correlation between the anti-S IgG titer before the infection and the intensity of the symptoms. However, this was not the case (Figure 4A). This finding again supports the conclusion that preexisting anti-S IgG levels correlate poorly with symptomatology during infections caused by Omicron variants. There was also no correlation between the COVID-19 symptoms and anti-S IgG titers after the infections (Figure 4B). Quite surprisingly, COVID-19 symptoms correlated with anti-N IgG levels detected after the infection (Spearman r −0.55, *p* = 0.03). Thus, individuals with lower anti-N IgG levels after infection were the ones who experienced the most intense COVID-19 symptoms (Figure 4C). This observation suggests that human anti-N IgG antibodies may play an important role in resolution of the disease.

## 4. Discussion

Studies worldwide have indicated that the levels of human IgG antibodies, particularly those reactive to the SARS-CoV-2 Spike protein, correlate with protection against novel infections and to mild and severe COVID-19 [46,47,48,49]. However, other studies suggest that human anti-S IgG levels wane over time and this may negatively influence protection [50,51]. Furthermore, it remains under debate to what extent the adaptive immunity response mounted against the original version of SARS-CoV-2 may confer protection against emerging viral variants, such as Omicron [49,50,51,52]. This manuscript provides insights into the symptomatology and anti-N and anti-S IgG levels before and after SARS-CoV-2 Omicron breakthrough infections in vaccinated individuals.

Notably, all except one participant exhibited robust anti-S IgG titers post-vaccination, indicating the effective priming of the immune system against antigens based on the original Wuhan SARS-CoV-2 strain. However, the emergence of SARS-CoV-2 Omicron variants challenged the efficacy of pre-existing immunity, leading to breakthrough infections despite high anti-S IgG titers detected in the participants prior to infections (Figure 1 and Figure S1). These data therefore imply that Omicron variants are well adapted to circumvent preexisting IgG reactive to the original virus. However, it is worth mentioning that other factors, not analyzed in this study, such as the number of immunizations events and previous infections, can affect the effectiveness of the IgG response to omicron variants.

Data obtained by others indicate that anti-S IgG levels correlate with protection against SARS-CoV-2 infections, with high IgG levels reducing the symptomatic infections and the possibility to develop severe disease [46,47,53]. Indeed, we observed that 41% of the detected infection events in our study were asymptomatic. On the other hand, we also noted that pre-existing anti-S IgG levels did not correlate with symptomatology (Figure 4B), nor were they able to fully prevent symptomatic infections by the Omicron variants (Table S2). Such observations have also been observed in other studies [54,55].

It is worth mentioning that our data are based on a limited number of individuals who were infected by a limited diversity of SARS-CoV-2 strains (Table S2), which may explain the differences in the results observed in other studies. Furthermore, our study is limited by the fact that we did not encounter any critical COVID-19 cases in our selected cohort of individuals. Another important limitation was that individuals self-scored symptom intensity.

One striking observation was the inverse correlation between symptom intensity and anti-N IgG levels (Figure 4A). Symptom variables and post-infection anti-N IgG titers were highly correlated (Figure 4A. Spearman r −0.55), suggesting that anti-N antibodies may play a role in symptom intensity. Even though our study had a limited cohort, another study, dealing with long COVID-19 cases and using a larger cohort, also observed a negative correlation between anti-N IgG and symptoms. Lower anti-N IgG titers were associated with longer symptom duration. On the other hand, higher anti-N IgG in the first week of SARS-CoV-2 RT-PCR positivity was associated with a shorter time for symptom resolution [56].

One important limitation of this study is the fact that T cell response was not analyzed. T-cell response is known to play an important role in combating SARS-CoV-2 infection against SARS-CoV-2 VOCs. Protective T cell epitopes are more broadly distributed and less mutated compared to those recognized by antibodies [36]. Hence, T-cell response is likely to play an important role in those individuals who were vaccinated and/or evolved hybrid immunity.

The possible relation between anti-N IgG and symptom resolution, which needs to be confirmed by further studies, provides novel insights into the relationship between the immune response and symptom intensity. These data suggest that a pre-mounted anti-N IgG response, induced by vaccination and/or natural infection, may be beneficial. Hence, our findings can help to guide novel vaccine formulations to include the Nucleocapsid along with the Spike antigen in the formulation.

In conclusion, this work provides an overview of Omicron breakthrough SARS-CoV-2 infections in vaccinated individuals, providing insights into immune response dynamics, variant susceptibility, and clinical outcomes, which can guide future strategies to combat COVID-19.

## Figures and Tables

**Table 1:** General data record of each individual. Data indicate COVID-19 test records and the sum of COVID-19 symptom intensity. The participant vaccination scheme is reported according to the initial letter of the vaccine’s name (A, for AstraZeneca, C for CoronaVac, J for Janssen, P for Pfizer). The anti-N and anti-S IgG titers before and after the infection event are reported. Omicron lineage was predicted based on the frequency of the local circulating lineage at the time of the infection. A * indicates that genomic information was experimentally obtained during this study (see text for details).

Individual Code	Age	Gender	Previous Infection	Positive Test	Sum of Symptoms	Vaccination Scheme before Infection	Omicron Sublineage	Anti-N IgG Titer before Infection	Anti-N IgG Titer after Infection	Anti-S IgG Titer before Infection	Anti-S IgG Titer after Infection
A	37	Female			0	PPP	BA.5	2	15	71	60
AA	41	Female		Yes	35	AAP	BA.2	5	59	44	66
B	32	Female			0	AA	BA.5	2	28	5	59
BB	19	Female			0	CCP	BA.2	6	97	76	50
C	46	Female			0	AAP	BA.2	3	17	36	65
CC	38	Female		Yes	28	AAP	BA.5	4	36	25	55
D	31	Female			0	CCP	BA.5	3	24	32	66
DD	41	Male			0	AAP	BA.5	4	24	36	38
E	51	Male			0	AAP	BA.5	3	18	94	84
G	36	Male		Yes	55	PPP	BA.5	3	23	28	80
GG	30	Female			0	PPA	BA.5	4	21	56	64
H	38	Male	Yes		0	AA	BA.2	6	17	15	59
I	44	Female		Yes	39	AAPP	BA.5	5	16	14	49
J	65	Female	Yes	Yes	22	CCPJ	BA.1	7	103	35	89
K	24	Female		Yes	33	PP	BA.1	7	70	43	62
L	61	Female		Yes	44	AAP	BA.2	5	20	48	84
M	31	Male		Yes	50	CCP	BA.5	8	20	43	89
N	44	Male		Yes	42	AAP	BA.2	13	25	42	55
O	74	Male		Yes	26	CCPJ	BA.1	9	76	17	85
P	39	Female		Yes	44	CCP	BA.2	5	76	41	56
Q	39	Female		Yes	38	AAA	BA.2	4	27	18	41
R	31	Female	Yes	Yes	66	PPPP	BA.5	4	55	37	43
S	39	Female	Yes	Yes	69	AAP	BA.2	4	19	41	70
U	41	Female			0	AAA	BA.5	4	33	25	65
V	24	Female		Yes	43	PP	BA.2	4	17	23	51
X	44	Male	Yes		0	AA	BA.5	10	89	48	82
Y	48	Female		Yes	42	AAP	BA.2	5	27	66	63

## Data Availability

The data supporting the findings of this study are available on request from the corresponding author (LFH).

## Supplementary Materials

The following supporting information can be downloaded at: https://www.mdpi.com/article/10.3390/vaccines12101149/s1, File: Supplementary information on SARS-CoV-2 genome sequencing of individual code N, Tables S1: Investigation form for coronavirus 2019; Table S2: General data record; Table S3: Frequency distribution table associated with the degree of intensity of COVID-19 symptoms; Figure S1: Time course of IgG titers and relevant events in each individual; Figure S2: Relative frequency of SARS-CoV-2 variants circulating in the Paraná state in Brazil and South America from 2020 to 2023; Figure S3: Descriptive flowchart of the clinical course of COVID-19 infection in cases related to individual N, representing the individuals’ contact history.

## References

[1] Iheanacho C.O., Eze U.I.H., Adida E.A. (2021). A systematic review of effectiveness of BNT162b2 mRNA and ChAdOx1 adenoviral vector COVID-19 vaccines in the general population. Bull. Natl. Res. Cent..

[2] Cerqueira-Silva T., Andrews J.R., Boaventura V.S., Ranzani O.T., Oliveira V.D.A., Paixão E.S., Júnior J.B., Machado T.M., Hitchings M.D.T., Dorion M. (2022). Effectiveness of CoronaVac, ChAdOx1 nCoV-19, BNT162b2, and Ad26.COV2.S among individuals with previous SARS-CoV-2 infection in Brazil: A test-negative, case-control study. Lancet Infect. Dis..

[3] Cerqueira-Silva T., Katikireddi S.V., Oliveira V.d.A., Flores-Ortiz R., Júnior J.B., Paixão E.S., Robertson C., Penna G.O., Werneck G.L., Barreto M.L. (2022). Vaccine effectiveness of heterologous CoronaVac plus BNT162b2 in Brazil. Nat. Med..

[4] Fadlyana E., Setiabudi D., Kartasasmita C.B., Putri N.D., Hadinegoro S.R., Mulholland K., Sofiatin Y., Suryadinata H., Hartantri Y., Sukandar H. (2023). Immunogenicity and safety in healthy adults of full dose versus half doses of COVID-19 vaccine (ChAdOx1-S or BNT162b2) or full-dose CoronaVac administered as a booster dose after priming with CoronaVac: A randomised, observer-masked, controlled trial in Indonesia. Lancet Infect. Dis..

[5] Voysey M., Clemens S.A.C., Madhi S.A., Weckx L.Y., Folegatti P.M., Aley P.K., Angus B., Baillie V.L., Barnabas S.L., Bhorat Q.E. (2021). Safety and efficacy of the ChAdOx1 nCoV-19 vaccine (AZD1222) against SARS-CoV-2: An interim analysis of four randomised controlled trials in Brazil, South Africa, and the UK. Lancet.

[6] Kahn R., Janusz C.B., Castro M.C., Matos A.d.R., Domingues C., Ponmattam J., Rey-Benito G., Toscano C.M., de Oliveira L.H., Rearte A. (2023). The effectiveness of COVID-19 vaccines in Latin America, 2021: A multicenter regional case-control study. Lancet Reg. Health Am..

[7] dos Santos C.V.B., Valiati N.C.M., de Noronha T.G., Porto V.B.G., Pacheco A.G., Freitas L.P., Coelho F.C., Gomes M.F.d.C., Bastos L.S., Cruz O.G. (2023). The effectiveness of COVID-19 vaccines against severe cases and deaths in Brazil from 2021 to 2022: A registry-based study. Lancet Reg. Health Am..

[8] Ravichandran S., Lee Y., Grubbs G., Coyle E.M., Klenow L., Akasaka O., Koga M., Adachi E., Saito M., Nakachi I. (2021). Longitudinal antibody repertoire in “mild” versus “severe” COVID-19 patients reveals immune markers associated with disease severity and resolution. Sci. Adv..

[9] Wu J., Liang B.-Y., Fang Y.-H., Wang H., Yang X.-L., Shen S., Chen L.-K., Li S.-M., Lu S.-H., Xiang T.-D. (2021). Occurrence of COVID-19 Symptoms During SARS-CoV-2 Infection Defines Waning of Humoral Immunity. Front. Immunol..

[10] Hajilooi M., Keramat F., Moazenian A., Rastegari-Pouyani M., Solgi G. (2023). The quantity and quality of anti-SARS-CoV-2 antibodies show contrariwise association with COVID-19 severity: Lessons learned from IgG avidity. Med. Microbiol. Immunol..

[11] Paula N.M., Conzentino M.S., Gonçalves A.C.A., da Silva R., Weissheimer K.V., Kluge C.H.S., Marins P.H.S.A., Camargo H.S.C., Farias L.R.P., Sant’ana T.P. (2023). Population-Based Analysis of the Immunoglobulin G Response to Different COVID-19 Vaccines in Brazil. Vaccines.

[12] Moreira E.D., Kitchin N., Xu X., Dychter S.S., Lockhart S., Gurtman A., Perez J.L., Zerbini C., Dever M.E., Jennings T.W. (2022). Safety and Efficacy of a Third Dose of BNT162b2 Covid-19 Vaccine. N. Engl. J. Med..

[13] Barin B., Kasap U., Selçuk F., Volkan E., Uluçkan Ö. (2022). Comparison of SARS-CoV-2 anti-spike receptor binding domain IgG antibody responses after CoronaVac, BNT162b2, ChAdOx1 COVID-19 vaccines, and a single booster dose: A prospective, longitudinal population-based study. Lancet Microbe.

[14] Pratesi F., Caruso T., Testa D., Tarpanelli T., Gentili A., Gioè D., Migliorini P. (2021). BNT162b2 mRNA SARS-CoV-2 Vaccine Elicits High Avidity and Neutralizing Antibodies in Healthcare Workers. Vaccines.

[15] Feikin D.R., Feikin D.R., Higdon M.M., Higdon M.M., Abu-Raddad L.J., Abu-Raddad L.J., Andrews N., Andrews N., Araos R., Araos R. (2022). Duration of effectiveness of vaccines against SARS-CoV-2 infection and COVID-19 disease: Results of a systematic review and meta-regression. Lancet.

[16] Ssentongo P., Ssentongo A.E., Voleti N., Groff D., Sun A., Ba D.M., Nunez J., Parent L.J., Chinchilli V.M., Paules C.I. (2022). SARS-CoV-2 vaccine effectiveness against infection, symptomatic and severe COVID-19: A systematic review and meta-analysis. BMC Infect. Dis..

[17] Shrotri M., Navaratnam A.M.D., Nguyen V., Byrne T., Geismar C., Fragaszy E., Beale S., Fong W.L.E., Patel P., Kovar J. (2021). Spike-antibody waning after second dose of BNT162b2 or ChAdOx1. Lancet.

[18] Levin E.G., Lustig Y., Cohen C., Fluss R., Indenbaum V., Amit S., Doolman R., Asraf K., Mendelson E., Ziv A. (2021). Waning Immune Humoral Response to BNT162b2 Covid-19 Vaccine over 6 Months. N. Engl. J. Med..

[19] Mao Y., Wang W., Ma J., Wu S., Sun F. (2022). Reinfection rates among patients previously infected by SARS-CoV-2: Systematic review and meta-analysis. Chin. Med. J..

[20] Hansen C.H., Michlmayr D., Gubbels S.M., Mølbak K., Ethelberg S. (2021). Assessment of protection against reinfection with SARS-CoV-2 among 4 million PCR-tested individuals in Denmark in 2020: A population-level observational study. Lancet.

[21] Nordström P., Ballin M., Nordström A. (2022). Risk of SARS-CoV-2 reinfection and COVID-19 hospitalisation in individuals with natural and hybrid immunity: A retrospective, total population cohort study in Sweden. Lancet Infect Dis..

[22] Chemaitelly H., Nagelkerke N., Ayoub H.H., Coyle P., Tang P., Yassine H.M., A Al-Khatib H., Smatti M.K., Hasan M.R., Al-Kanaani Z. (2022). Duration of immune protection of SARS-CoV-2 natural infection against reinfection. J. Travel. Med..

[23] Schmidt F., Weisblum Y., Rutkowska M., Poston D., DaSilva J., Zhang F., Bednarski E., Cho A., Schaefer-Babajew D.J., Gaebler C. (2021). High genetic barrier to SARS-CoV-2 polyclonal neutralizing antibody escape. Nature.

[24] Wratil P.R., Stern M., Priller A., Willmann A., Almanzar G., Vogel E., Feuerherd M., Cheng C.-C., Yazici S., Christa C. (2022). Three exposures to the spike protein of SARS-CoV-2 by either infection or vaccination elicit superior neutralizing immunity to all variants of concern. Nat. Med..

[25] Andrews N., Stowe J., Kirsebom F., Toffa S., Rickeard T., Gallagher E., Gower C., Kall M., Groves N., O’Connell A.M. (2022). Covid-19 Vaccine Effectiveness against the Omicron (B.1.1.529) Variant. N. Engl. J. Med..

[26] Altarawneh H.N., Chemaitelly H., Ayoub H.H., Tang P., Hasan M.R., Yassine H.M., Al-Khatib H.A., Smatti M.K., Coyle P., Al-Kanaani Z. (2022). Effects of Previous Infection and Vaccination on Symptomatic Omicron Infections. N. Engl. J. Med..

[27] Freire-Neto F.P., Teixeira D.G., da Cunha D.C.S., Morais I.C., Tavares C.P.M., Gurgel G.P., Medeiros S.D.N., dos Santos D.C., Sales A.D.O., Jeronimo S.M.B. (2022). SARS-CoV-2 reinfections with BA.1 (Omicron) variant among fully vaccinated individuals in northeastern Brazil. PLoS Negl. Trop. Dis..

[28] Oliveira J.R., Ruiz C.M.R., Machado R.R.G., Magawa J.Y., Daher I.P., Urbanski A.H., Schmitz G.J.H., Arcuri H.A., Ferreira M.A., Sasahara G.L. (2023). Immunodominant antibody responses directed to SARS-CoV-2 hotspot mutation sites and risk of immune escape. Front. Immunol..

[29] Rhoden J., Hoffmann A.T., Stein J.F., da Silva M.S., Gularte J.S., Filippi M., Demoliner M., Girardi V., Spilki F.R., Fleck J.D. (2024). Diversity of Omicron sublineages and clinical characteristics in hospitalized patients in the southernmost state of Brazil. BMC Infect. Dis..

[30] Sette A., Crotty S. (2022). Immunological memory to SARS-CoV-2 infection and COVID-19 vaccines. Immunol. Rev..

[31] Goel R.R., Apostolidis S.A., Painter M.M., Mathew D., Pattekar A., Kuthuru O., Gouma S., Hicks P., Meng W., Rosenfeld A.M. (2021). Distinct antibody and memory B cell responses in SARS-CoV-2 naïve and recovered individuals following mRNA vaccination. Sci. Immunol..

[32] Otto S.P., Day T., Arino J., Colijn C., Dushoff J., Li M., Mechai S., Van Domselaar G., Wu J., Earn D.J. (2021). The origins and potential future of SARS-CoV-2 variants of concern in the evolving COVID-19 pandemic. Curr. Biol..

[33] Calistri P., Amato L., Puglia I., Cito F., Di Giuseppe A., Danzetta M.L., Morelli D., Di Domenico M., Caporale M., Scialabba S. (2021). Infection sustained by lineage B.1.1.7 of SARS-CoV-2 is characterised by longer persistence and higher viral RNA loads in nasopharyngeal swabs. Int. J. Infect. Dis..

[34] Zhang D., Zhong J., Xiong H., Li Y., Guo T., Peng B., Fang C., Kang Y., Tan J., Ma Y. (2023). Protective Effect of Inactivated COVID-19 Vaccines against Omicron BA.2 Infection in Guangzhou: A Test-Negative Case-Control Real-World Study. Vaccines.

[35] Garrett N., Tapley A., Andriesen J., Seocharan I., Fisher L.H., Bunts L., Espy N., Wallis C.L., Randhawa A.K., Ketter N. (2022). High Rate of Asymptomatic Carriage Associated with Variant Strain Omicron. medRxiv..

[36] Carabelli A.M., Peacock T.P., Thorne L.G., Harvey W.T., Hughes J., Peacock S.J., Barclay W.S., de Silva T.I., Towers G.J., COVID-19 Genomics UK Consortium (2023). SARS-CoV-2 variant biology: Immune escape, transmission and fitness. Nat. Rev. Microbiol..

[37] Markov P.V., Ghafari M., Beer M., Lythgoe K., Simmonds P., Stilianakis N.I., Katzourakis A. (2023). The evolution of SARS-CoV-2. Nat. Rev. Microbiol..

[38] Tuekprakhon A., Nutalai R., Dijokaite-Guraliuc A., Zhou D., Ginn H.M., Selvaraj M., Liu C., Mentzer A.J., Supasa P., Duyvesteyn H.M. (2022). Antibody escape of SARS-CoV-2 Omicron BA.4 and BA.5 from vaccine and BA.1 serum. Cell.

[39] Huergo L.F., Paula N.M., Gonçalves A.C.A., Kluge C.H.S., Marins P.H.S.A., Camargo H.S.C., Sant’ana T.P., Farias L.R.P., Aldrighi J.D., Lima S. (2022). SARS-CoV-2 Seroconversion in Response to Infection and Vaccination: A Time Series Local Study in Brazil. Microbiol. Spectr..

[40] Conzentino M.S., Gonçalves A.C.A., Paula N.M., Rego F.G.M., Zanette D.L., Aoki M.N., Nardin J.M., Huergo L.F. (2022). A magnetic bead immunoassay to detect high affinity human IgG reactive to SARS-CoV-2 Spike S1 RBD produced in *Escherichia coli*. Braz. J. Microbiol..

[41] Conzentino M.S., Santos T.P., Selim K.A., Wagner B., Alford J.T., Deobald N., Paula N.M., Rego F.G., Zanette D.L., Aoki M.N. (2021). Ultra-fast, high throughput and inexpensive detection of SARS-CoV-2 seroconversion using Ni^2+^ magnetic beads. Anal. Biochem..

[42] Huergo L.F., Selim K.A., Conzentino M.S., Gerhardt E.C.M., Santos A.R.S., Wagner B., Alford J.T., Deobald N., Pedrosa F.O., de Souza E.M. (2021). Magnetic Bead-Based Immunoassay Allows Rapid, Inexpensive, and Quantitative Detection of Human SARS-CoV-2 Antibodies. ACS Sens..

[43] Conzentino M.S., Forchhammer K., Souza E.M., Pedrosa F.O., Nogueira M.B., Raboni S.M., Rego F.G.M., Zanette D.L., Aoki M.N., Nardin J.M. (2021). Antigen production and development of an indirect ELISA based on the nucleocapsid protein to detect human SARS-CoV-2 seroconversion. Braz. J. Microbiol..

[44] Bochnia-Bueno L., De Almeida S.M., Raboni S.M., Adamoski D., Amadeu L.L.M., Carstensen S., Nogueira M.B. (2022). Dynamic of humoral response to SARS-CoV-2 anti-Nucleocapsid and Spike proteins after CoronaVac vaccination. Diagn. Microbiol. Infect. Dis..

[45] Rambaut A., Holmes E.C., O’Toole Á., Hill V., McCrone J.T., Ruis C., du Plessis L., Pybus O.G. (2020). A dynamic nomenclature proposal for SARS-CoV-2 lineages to assist genomic epidemiology. Nat. Microbiol..

[46] Wei J., Pouwels K.B., Stoesser N., Matthews P.C., Diamond I., Studley R., Rourke E., Cook D., Bell J.I., Newton J.N. (2022). Antibody responses and correlates of protection in the general population after two doses of the ChAdOx1 or BNT162b2 vaccines. Nat. Med..

[47] Gilbert P.B., Montefiori D.C., McDermott A.B., Fong Y., Benkeser D., Deng W., Zhou H., Houchens C.R., Martins K., Jayashankar L. (2022). Immune correlates analysis of the mRNA-1273 COVID-19 vaccine efficacy clinical trial. Science.

[48] Yamamoto S., Mizoue T., Ohmagari N. (2023). Analysis of Previous Infection, Vaccinations, and Anti-SARS-CoV-2 Antibody Titers and Protection Against Infection With the SARS-CoV-2 Omicron BA.5 Variant. JAMA Netw. Open..

[49] Murray S.M., Ansari A.M., Frater J., Klenerman P., Dunachie S., Barnes E., Ogbe A. (2023). The impact of pre-existing cross-reactive immunity on SARS-CoV-2 infection and vaccine responses. Nat. Rev. Immunol..

[50] Yang Z.-R., Jiang Y.-W., Li F.-X., Liu D., Lin T.-F., Zhao Z.-Y., Wei C., Jin Q.-Y., Li X.-M., Jia Y.-X. (2023). Efficacy of SARS-CoV-2 vaccines and the dose-response relationship with three major antibodies: A systematic review and meta-analysis of randomised controlled trials. Lancet Microbe.

[51] Buhre J.S., Pongracz T., Künsting I., Lixenfeld A.S., Wang W., Nouta J., Lehrian S., Schmelter F., Lunding H.B., Dühring L. (2023). mRNA vaccines against SARS-CoV-2 induce comparably low long-term IgG Fc galactosylation and sialylation levels but increasing long-term IgG4 responses compared to an adenovirus-based vaccine. Front. Immunol..

[52] Huang C.Q., Vishwanath S., Carnell G.W., Chan A.C.Y., Heeney J.L. (2023). Immune imprinting and next-generation coronavirus vaccines. Nat. Microbiol..

[53] Kang H., Jung J., Ko G.Y., Lee J., Oh E.-J. (2024). Evaluation of Long-Term Adaptive Immune Responses Specific to SARS-CoV-2: Effect of Various Vaccination and Omicron Exposure. Vaccines.

[54] Goguet E., Olsen C.H., Meyer W.A., Ansari S., Powers J.H., Conner T.L., Coggins S.A., Wang W., Wang R., Illinik L. (2024). Immune and behavioral correlates of protection against symptomatic post-vaccination SARS-CoV-2 infection. Front. Immunol..

[55] Spiteri G., D’agostini M., Abedini M., Ditano G., Collatuzzo G., Boffetta P., Vimercati L., Sansone E., De Palma G., Modenese A. (2024). Protective role of SARS-CoV-2 anti-S IgG against breakthrough infections among European healthcare workers during pre and post-Omicron surge-ORCHESTRA project. Infection.

[56] Jia X., Cao S., Lee A.S., Manohar M., Sindher S.B., Ahuja N., Artandi M., Blish C.A., Blomkalns A.L., Chang I. (2022). Anti-nucleocapsid antibody levels and pulmonary comorbid conditions are linked to post-COVID-19 syndrome. JCI Insight.

