# Supplementary material for: Symptomatology and IgG Levels before and after SARS-CoV-2 Omicron Breakthrough Infections in Vaccinated Individuals
Source: Vaccines (Basel). 2024 Oct 8;12(10):1149. doi: 10.3390/vaccines12101149 (PMC11512233; doi:10.3390/vaccines12101149)
Supplement: Supplementary file 1 [file vaccines-12-01149-s001.zip › vaccines-3099876-supplementary.pdf]

SUPPLEMENTARY MATERIAL

File:

Suplementary information on SARS-CoV-2 genome sequencing of individual code N

Illumina sequence raw data was processed using the COVID Lineage pipeline on the Dragen analysis platform. After quality control, the consensus sequence was assembly and mapped against the reference strain (NC_045512.2) to identity mutations, which are described below:

Nucleotide substitutions

C241T,T670G,C2790T,C3037T,G4184A,C4321T,C6354T,C9344T,A9424G,C9534T,C9866T,C10029T,C10198T,G10447A,C10449A,C12880T,C14408T,C15714T,C17410T,A18163G,C19955T,A20055G,C21618T,G21987A,T22200G,G22578A,C22674T,T22679C,C22686T,A22688G,G22775A,A22786C,G22813T,T22882G,G22992A,C22995A,A23013C,A23040G,A23055G,A23063T,T23075C,A23403G,C23525T,T23599G,C23604A,C23854A,G23948T,A24424T,T24469A,C25000T,C25584T,A26053G,C26060T,C26270T,G26709A,C26858T,A27259C,G27382C,A27383T,T27384C,C27807T,A28271T,C28311T,G28881A,G28882A,G28883C,A29510C,T29834A

Amino acid substitutios

E:T9I,M:A63T,N:P13L,N:R203K,N:G204R,N:S413R,ORF1a:S135R,ORF1a:T842I,ORF1a:G1307S,ORF1a:S2030L,ORF1a:L3027F,ORF1a:T3090I,ORF1a:L3201F,ORF1a:T3255I,ORF1a:P3395H,ORF1b:P314L,ORF1b:R1315C,ORF1b:I1566V,ORF1b:T2163I,ORF3a:T221A,ORF3a:T223I,ORF6:D61L,ORF9b:P10S,S:T19I,S:A27S,S:G142D,S:V213G,S:G339D,S:S371F,S:S373P,S:S375F,S:T376A,S:D405N,S:R408S,S:K417N,S:N440K,S:S477N,S:T478K,S:E484A,S:Q493R,S:Q498R,S:N501Y,S:Y505H,S:D614G,S:H655Y,S:N679K,S:P681H,S:N764K,S:D796Y,S:Q954H,S:N969K

Amino acid deletion

N:E31-,N:R32-,N:S33-,ORF1a:S3675-,ORF1a:G3676-,ORF1a:F3677-,ORF9b:E27-,ORF9b:N28-,ORF9b:A29-,S:L24-,S:P25-,S:P26-

Table S1 - INVESTIGATION FORM FOR CORONAVIRUS 2019

Notification State (UF):

Notification Municipality:

Identification

Full name:

Data of Birth:

Coountry of Origin:

Gender:
| |Male
| |Female

Contact Phone:

Clinical Data

Notification Date:

Onset of Symptoms Date:

Symptoms: (Mark X)
| |Asymptomatic | |Fever | |Sore Throat | |Shortness of Breath | |Cough | | Runny Nose
| |Headache | | Taste Disorders | | Smell Disorders | |Others

Conditions: (Mark X)
| |Age above 60 years | |Advanced-stage chronic kidney diseases (Stages 3, 4, and 5) | |Carrier of chromosomal diseases or immunological fragility state | |Chronic heart diseases | |Chronic liver diseases | |Chronic neurological diseases | |Diabetes | |Hematologic diseases (e.g., sickle cell anemia, thalassemia major) | |Immunocompromised
| |Obesity (BMI ≥ 30) | |Postpartum (up to 45 days after delivery) | |Pregnant | |Uncontrolled chronic respiratory diseases

**Symptoms**	**Symptoms intensity**										
	**1**	**2**	**3**	**4**	**5**	**6**	**7**	**8**	**9**	**10**	**Observations:**
**Fever or Chills**											
**Sore Throat**											
**Shortness of Breath**											
**Cough**											
**Headache**											
**Congestion or Runny Nose**											
**Loss of Smell or Taste**											
**Muscle or Body aches**											
**Diarrhea or Nausea**											
**Others:**											

Positive teste for SARS-CoV-2?
|__| Yes |__|No

Type of test: |__| RT-PCR |__| Rapid Antigen Test
|__| Rapid Antibody Test |__|ELISA |__|ECLIA |__|CLIA

Collection Date:_|____|____

Test Result: |__| Positive |___|Negative |___|Inconclusive

Table S1: Investigation Form for Coronavirus 2019: Symptoms, Tests, Monitoring and Identification of Risk Conditions.

Table S2

Individual Code	Age	Gender	Underlying health conditions	Previous Infection	Positive test	Fever or chills	Sore throat	Shortness of breath	Cough	Headache	Congestion runny nose	Loss of taste or smell	Muscle or body aches	Diarrhea or Nausea	Sum of symptoms	Vaccination Scheme Before Infecction	Likely Omicron sublineage	IgG N titer Median (95% CI)	Anti-N IgG titer before infection	Anti-N IgG titer after infection	IgG S titer Median (95% CI)	Anti-S IgG titer before infection	Anti-S IgG titer after infection
A	37	Female				0	0	0	0	0	0	0	0	0	0	PPP	BA.5	2 (94%)	2	15	36 (88%)	71	60
AA	41	Female			Yes	9	2	2	9	0	3	2	8	0	35	AAP	BA.2	7 (75%)	5	59	51 (75%)	44	66
B	32	Female				0	0	0	0	0	0	0	0	0	0	AA	BA.5	3 (96%)	2	28	7.5 (99%)	5	59
BB	19	Female				0	0	0	0	0	0	0	0	0	0	CCP	BA.2	56 (75%)	6	97	50 (75%)	76	50
C	46	Female				0	0	0	0	0	0	0	0	0	0	AAP	BA.2	10 (94%)	3	17	48 (94%)	36	65
CC	38	Female			Yes	8	0	0	5	0	3	5	7	0	28	AAP	BA.5	7.5 (88%)	4	36	22 (88%)	25	55
D	31	Female				0	0	0	0	0	0	0	0	0	0	CCP	BA.5	3 (94%)	3	24	49 (94%)	32	66
DD	41	Male				0	0	0	0	0	0	0	0	0	0	AAP	BA.5	5 (75%)	4	24	38 (75%)	36	38
E	51	Male				0	0	0	0	0	0	0	0	0	0	AAP	BA.5	5.5 (96%)	3	18	22 (99%)	94	84
G	36	Male			Yes	9	8	7	9	3	10	9	0	0	55	PPP	BA.5	3 (94%)	3	23	50 (94%)	28	80
GG	30	Female				0	0	0	0	0	0	0	0	0	0	PPA	BA.5	4 (75%)	4	21	56 (75%)	56	64
H	38	Male		Yes		0	0	0	0	0	0	0	0	0	0	AA	BA.2	7 (99%)	6	17	24 (98%)	15	59
I	44	Female			Yes	6	5	2	10	6	1	6	2	1	39	AAPP	BA.5	8 (96%)	5	16	57 (97%)	14	49
J	65	Female	Age above 60 years	Yes	Yes	4	0	0	3	0	3	8	4	0	22	CCPJ	BA.1	12 (88%)	7	103	43 (88%)	35	89
K	24	Female			Yes	8	9	0	3	5	5	0	3	0	33	PP	BA.1	9.5 (99%)	7	70	60 (99%)	43	62
L	61	Female	Age above 60 years		Yes	9	9	1	9	3	3	6	3	1	44	AAP	BA.2	4 (94%)	5	20	30 (88%)	48	84
M	31	Male			Yes	8	10	1	4	8	5	5	9	0	50	CCP	BA.5	6 (97%)	8	20	74 (97%)	43	89
N	44	Male			Yes	9	9	1	9	3	2	3	5	1	42	AAP	BA.2	3.5 (97%)	13	25	34 (97%)	42	55
O	74	Male	Age above 60 years		Yes	9	0	0	0	0	9	3	5	0	26	CCPJ	BA.1	23 (88%)	9	76	76 (88%)	17	85
P	39	Female			Yes	9	9	1	9	3	3	6	3	1	44	CCP	BA.2	6 (99%)	5	76	30 (99%)	41	56
Q	39	Female			Yes	9	8	1	6	6	1	6	0	1	38	AAA	BA.2	5 (94%)	4	27	21 (94%)	18	41
R	31	Female	Immunocompromised	Yes	Yes	10	9	8	9	7	9	7	7	0	66	PPPP	BA.5	8 (97%)	4	55	69 (96%)	37	43
S	39	Female	Obesity	Yes	Yes	10	10	10	10	3	10	8	7	1	69	AAP	BA.2	4 (96%)	4	19	66 (98%)	41	70
U	41	Female				0	0	0	0	0	0	0	0	0	0	AAA	BA.5	7 (94%)	4	33	24 (88%)	25	65
V	24	Female			Yes	0	10	10	5	0	0	0	10	8	43	PP	BA.2	5 (99%)	4	17	34 (99%)	23	51
X	44	Male		Yes		0	0	0	0	0	0	0	0	0	0	AA	BA.5	16 (88%)	10	89	53 (88%)	48	82
Y	48	Female			Yes	9	9	1	9	3	2	3	5	1	42	AAP	BA.2	8 (75%)	5	27	63 (75%)	66	63

Table S2: General data record. Indicating test records and onset of symptoms, intensity of COVID-19 symptoms (evaluated on a scale of 0 to 10) and sum symptoms. Participants' vaccination scheme is reported according to the initial letter of the vaccine's name (A, for AstraZeneca, C for CoronaVac, J for Janssen, P for Pfizer). The anti-N and anti-S IgG titers before and after the infection event are reported.

Table S3

Symptomatology	Mean ± SD	Frequency Distribution (%) - Degree of intensity of symptoms COVID-19 (from 0 to 10)										
		**0**	**1**	**2**	**3**	**4**	**5**	**6**	**7**	**8**	**9**	**10**
**Fever or chills**	7,9 ± 2,6	6%	0%	0%	0%	6%	0%	6%	0%	19%	50%	13%
**Sore throat**	6,7 ± 3,9	19%	0%	6%	0%	0%	6%	0%	0%	13%	38%	19%
**Shortness of breath**	2,8 ± 3,7	25%	38%	13%	0%	0%	0%	0%	6%	6%	0%	13%
**Cough**	6,8 ± 3,1	6%	0%	0%	13%	6%	13%	6%	0%	0%	44%	13%
**Headache**	3,1 ± 2,7	31%	0%	0%	38%	0%	6%	13%	6%	6%	0%	0%
**Congestion or runny nose**	4,3 ± 3,4	6%	13%	13%	31%	0%	13%	0%	0%	0%	13%	13%
**Loss of taste or smell**	4,8 ± 2,7	13%	0%	6%	19%	0%	13%	25%	6%	13%	6%	0%
**Muscle or body aches**	4,9 ± 3,0	13%	0%	6%	19%	6%	19%	0%	19%	6%	6%	6%
**Diarrhea or Nausea**	0,9 ± 1,9	50%	44%	0%	0%	0%	0%	0%	0%	6%	0%	0%

Table S3: Frequency distribution table associated with the degree of intensity of COVID-19 symptoms. Note: based on the latest update from the Ministry of Health (September 2021), primary symptoms of COVID-19 have been incorporated into the table. SD: Standard Deviation.

Figure S1 pages 5 - 11

A

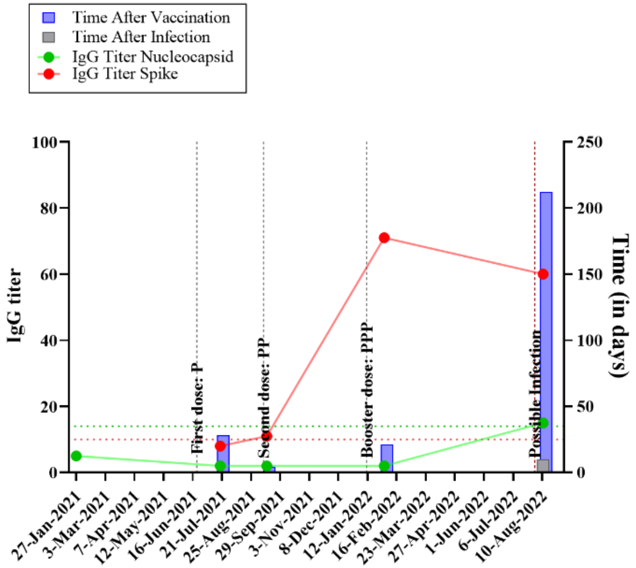

AA

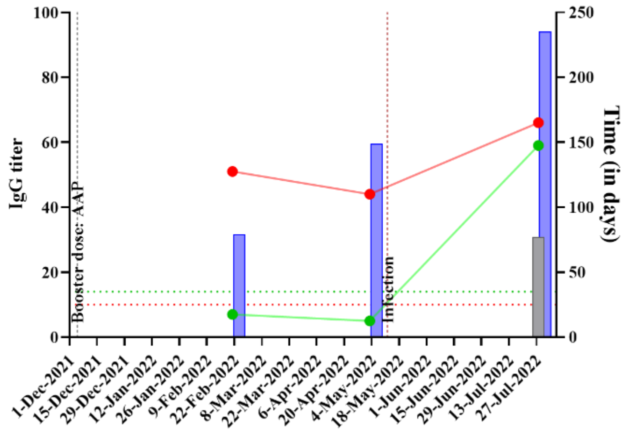

B

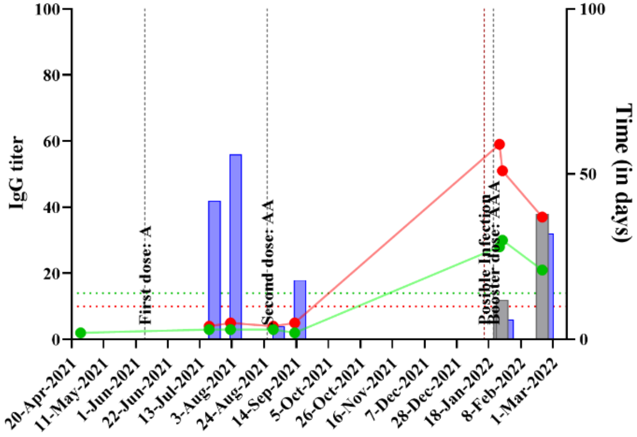

BB

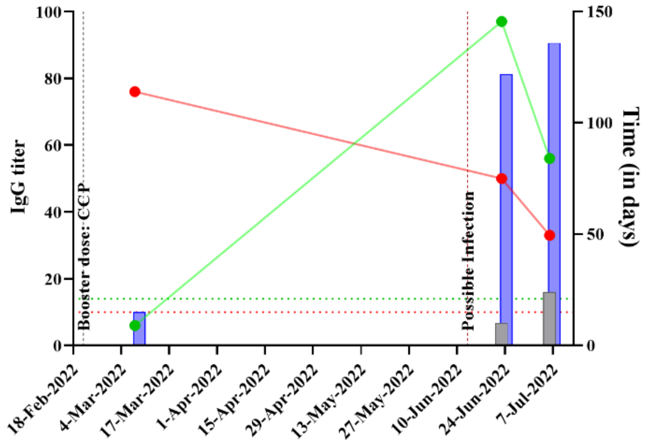

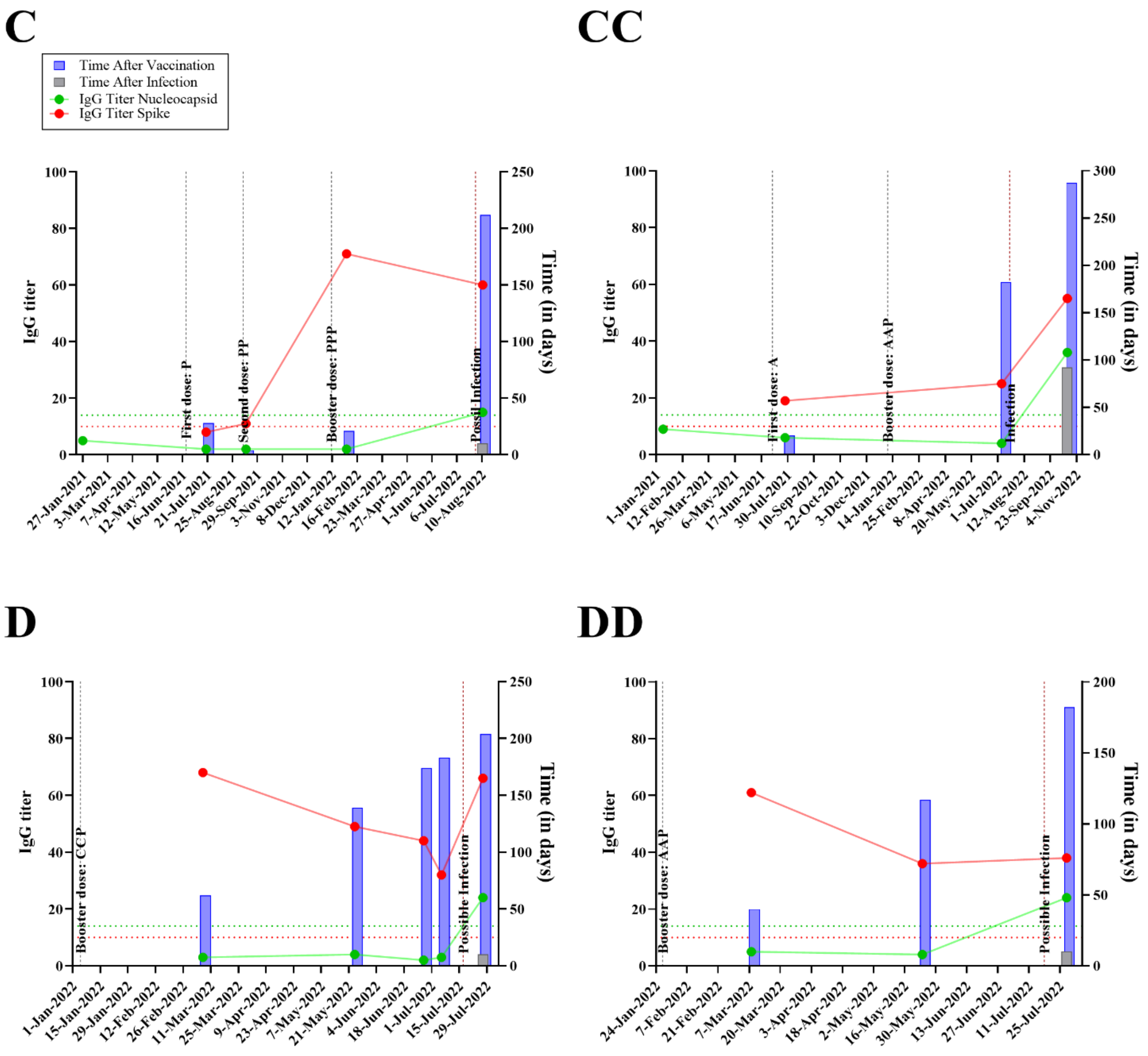
C
CC
D
DD
Time After Vaccination
Time After Infection
IgG Titer Nucleocapsid
IgG Titer Spike
IgG titer
Time (in days)
First dose: P
Second dose: PP
Booster dose: PPP
Possil Infection
First dose: A
Booster dose: AAP
Infection
Booster dose: CCP
Possible Infection
Booster dose: AAP
Possible Infection

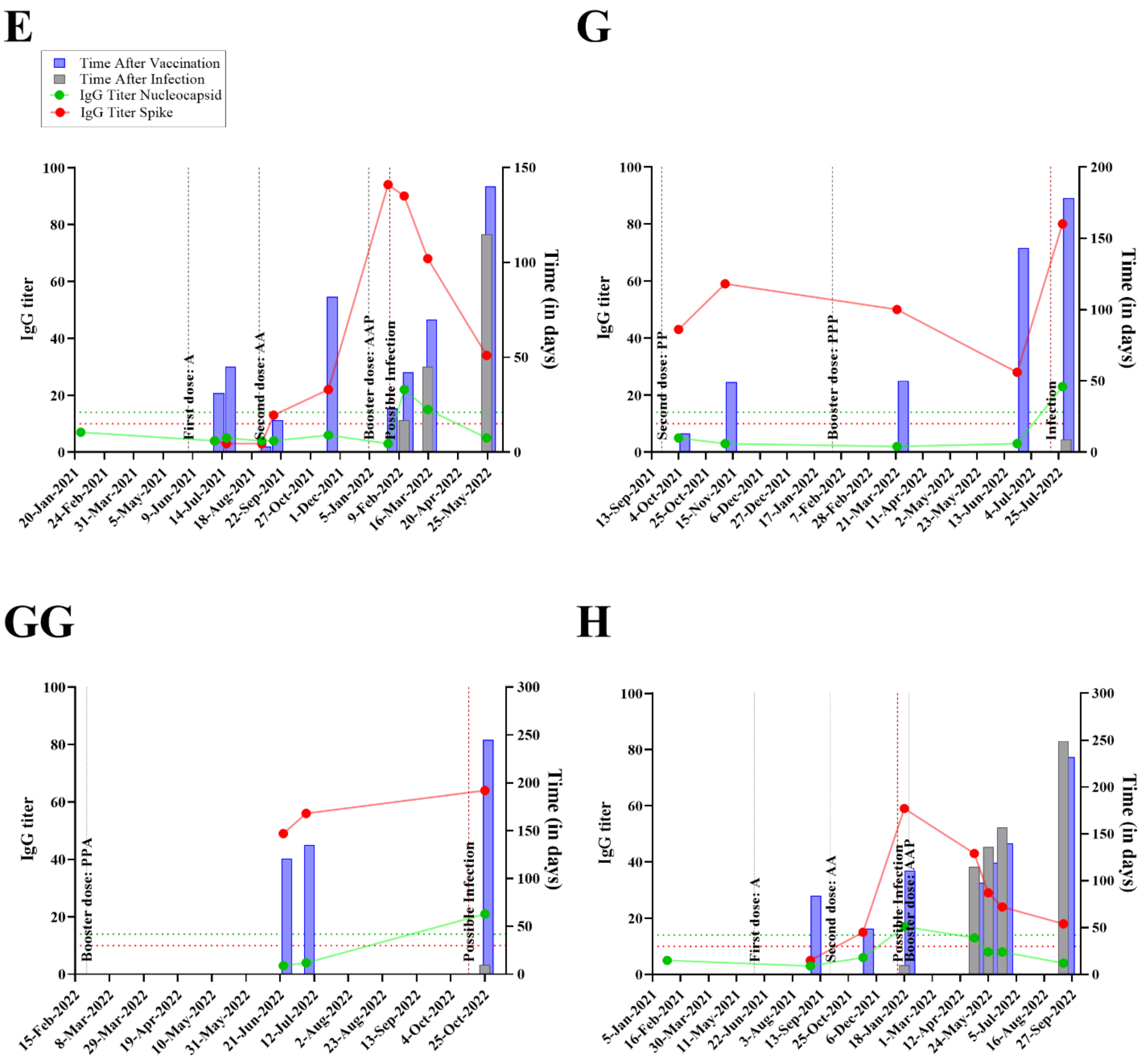
E
Time After Vaccination
Time After Infection
IgG Titer Nucleocapsid
IgG Titer Spike
IgG titer
Time (in days)
First dose: A
Second dose: AA
Booster dose: AAP
Possible Infection
20-Jan-2021
24-Feb-2021
31-Mar-2021
5-May-2021
9-Jun-2021
14-Jul-2021
18-Aug-2021
22-Sep-2021
27-Oct-2021
1-Dec-2021
5-Jan-2022
9-Feb-2022
16-Mar-2022
20-Apr-2022
25-May-2022
G
IgG titer
Time (in days)
Second dose: PP
Booster dose: PPP
Infection
13-Sep-2021
4-Oct-2021
25-Oct-2021
15-Nov-2021
6-Dec-2021
27-Dec-2021
17-Jan-2022
7-Feb-2022
28-Feb-2022
21-Mar-2022
11-Apr-2022
2-May-2022
23-May-2022
13-Jun-2022
4-Jul-2022
25-Jul-2022
GG
IgG titer
Time (in days)
Booster dose: PPA
Possible Infection
15-Feb-2022
8-Mar-2022
29-Mar-2022
19-Apr-2022
10-May-2022
31-May-2022
21-Jun-2022
12-Jul-2022
2-Aug-2022
23-Aug-2022
13-Sep-2022
4-Oct-2022
25-Oct-2022
H
IgG titer
Time (in days)
First dose: A
Second dose: AA
Possible Infection
Booster dose: AAP
5-Jan-2021
16-Feb-2021
30-Mar-2021
11-May-2021
22-Jun-2021
3-Aug-2021
13-Sep-2021
25-Oct-2021
6-Dec-2021
18-Jan-2022
1-Mar-2022
12-Apr-2022
24-May-2022
5-Jul-2022
16-Aug-2022
27-Sep-2022

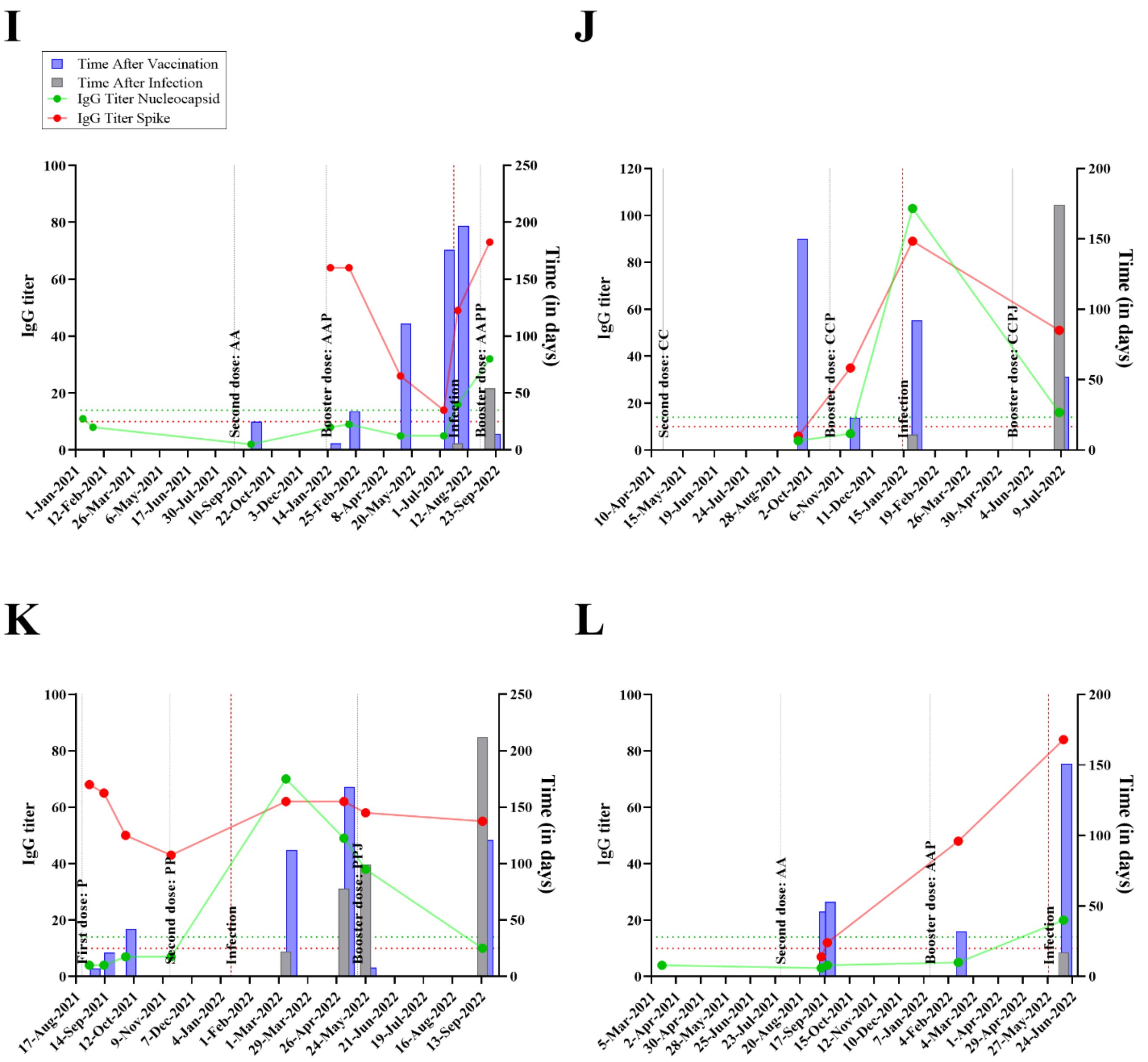
I
J
K
L
Time After Vaccination
Time After Infection
IgG Titer Nucleocapsid
IgG Titer Spike
IgG titer
Time (in days)
Second dose: AA
Booster dose: AAP
Infection
Booster dose: AAPP
1-Jan-2021
12-Feb-2021
26-Mar-2021
6-May-2021
17-Jun-2021
30-Jul-2021
10-Sep-2021
22-Oct-2021
3-Dec-2021
14-Jan-2022
25-Feb-2022
8-Apr-2022
20-May-2022
1-Jul-2022
12-Aug-2022
23-Sep-2022
Second dose: CC
Booster dose: CCP
Infection
Booster dose: CCPJ
10-Apr-2021
15-May-2021
19-Jun-2021
24-Jul-2021
28-Aug-2021
2-Oct-2021
6-Nov-2021
11-Dec-2021
15-Jan-2022
19-Feb-2022
26-Mar-2022
30-Apr-2022
4-Jun-2022
9-Jul-2022
First dose: P
Second dose: PP
Infection
Booster dose: PPJ
17-Aug-2021
14-Sep-2021
12-Oct-2021
9-Nov-2021
7-Dec-2021
4-Jan-2022
1-Feb-2022
1-Mar-2022
29-Mar-2022
26-Apr-2022
24-May-2022
21-Jun-2022
19-Jul-2022
16-Aug-2022
13-Sep-2022
Second dose: AA
Booster dose: AAP
Infection
5-Mar-2021
2-Apr-2021
30-Apr-2021
28-May-2021
25-Jun-2021
23-Jul-2021
20-Aug-2021
17-Sep-2021
15-Oct-2021
12-Nov-2021
10-Dec-2021
7-Jan-2022
4-Feb-2022
4-Mar-2022
1-Apr-2022
29-Apr-2022
27-May-2022
24-Jun-2022

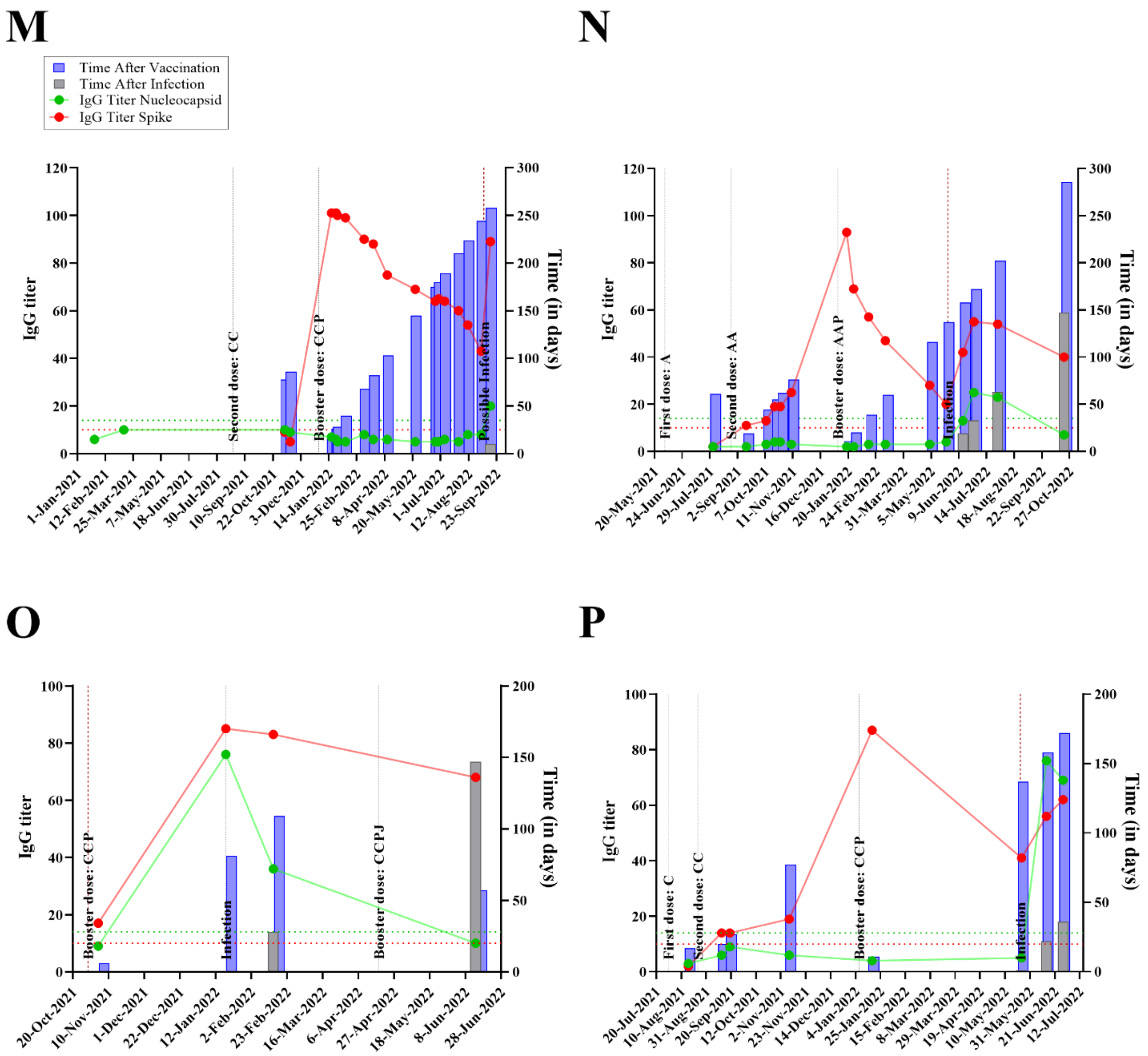
M
N
O
P
Time After Vaccination
Time After Infection
IgG Titer Nucleocapsid
IgG Titer Spike
IgG titer
Time (in days)
Second dose: CC
Booster dose: CCP
Possible Infection
First dose: A
Second dose: AA
Booster dose: AAP
Infection
Booster dose: CCP
Infection
Booster dose: CCPJ
First dose: C
Second dose: CC
Booster dose: CCP
Infection
1-Jan-2021
12-Feb-2021
25-Mar-2021
7-May-2021
18-Jun-2021
30-Jul-2021
10-Sep-2021
22-Oct-2021
3-Dec-2021
14-Jan-2022
25-Feb-2022
8-Apr-2022
20-May-2022
1-Jul-2022
12-Aug-2022
23-Sep-2022
20-May-2021
24-Jun-2021
29-Jul-2021
2-Sep-2021
7-Oct-2021
11-Nov-2021
16-Dec-2021
20-Jan-2022
24-Feb-2022
31-Mar-2022
5-May-2022
9-Jun-2022
14-Jul-2022
18-Aug-2022
22-Sep-2022
27-Oct-2022
20-Oct-2021
10-Nov-2021
1-Dec-2021
22-Dec-2021
12-Jan-2022
2-Feb-2022
23-Feb-2022
16-Mar-2022
6-Apr-2022
27-Apr-2022
18-May-2022
8-Jun-2022
28-Jun-2022
20-Jul-2021
10-Aug-2021
31-Aug-2021
20-Sep-2021
12-Oct-2021
2-Nov-2021
23-Nov-2021
14-Dec-2021
4-Jan-2022
25-Jan-2022
15-Feb-2022
8-Mar-2022
29-Mar-2022
19-Apr-2022
10-May-2022
31-May-2022
21-Jun-2022
12-Jul-2022

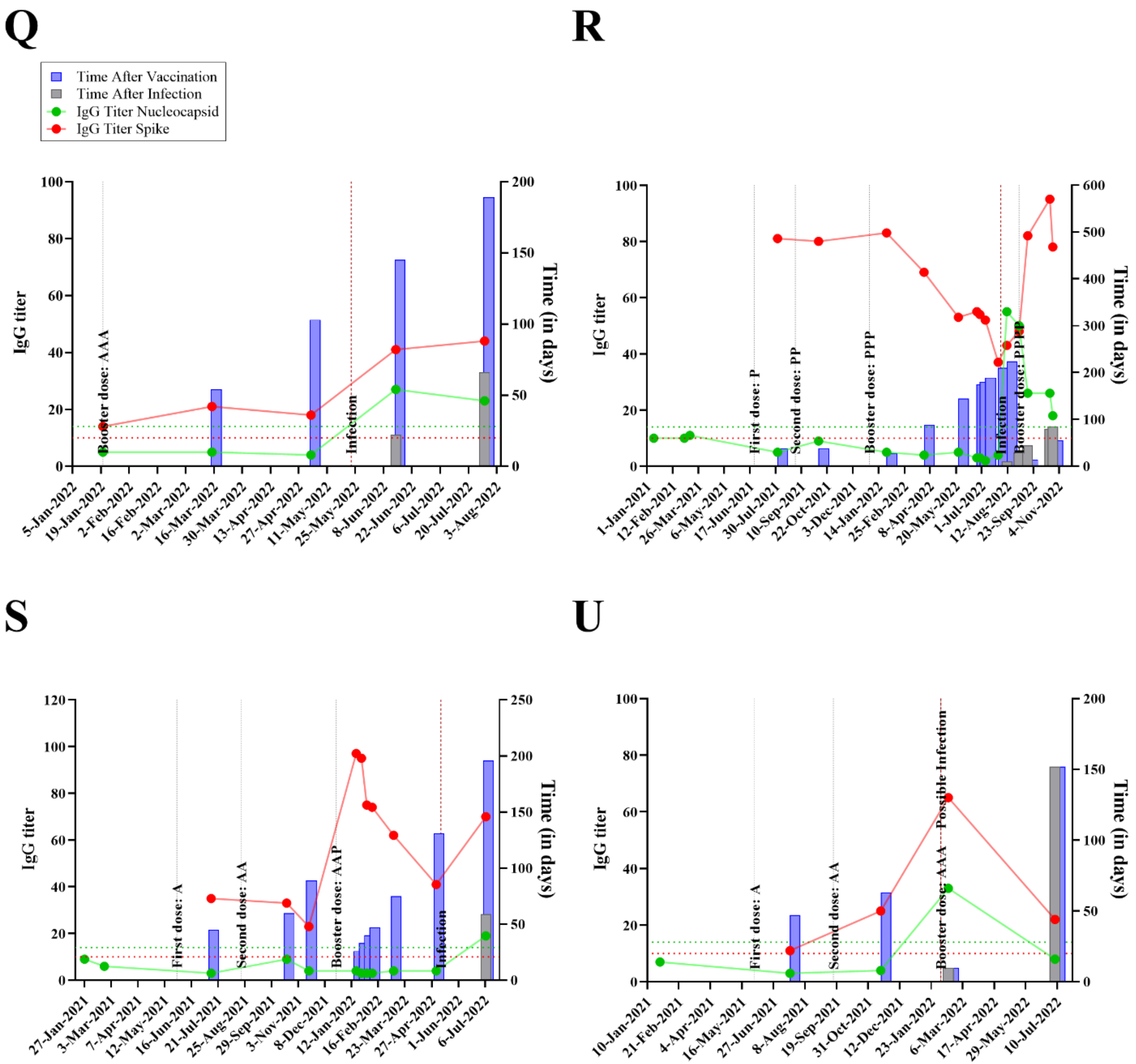
Q
R
S
U
Time After Vaccination
Time After Infection
IgG Titer Nucleocapsid
IgG Titer Spike
IgG titer
Time (in days)
Booster dose: AAA
Infection
First dose: P
Second dose: PP
Booster dose: PPP
Booster dose: PPPP
First dose: A
Second dose: AA
Booster dose: AAP
Booster dose: AAA
Possible Infection

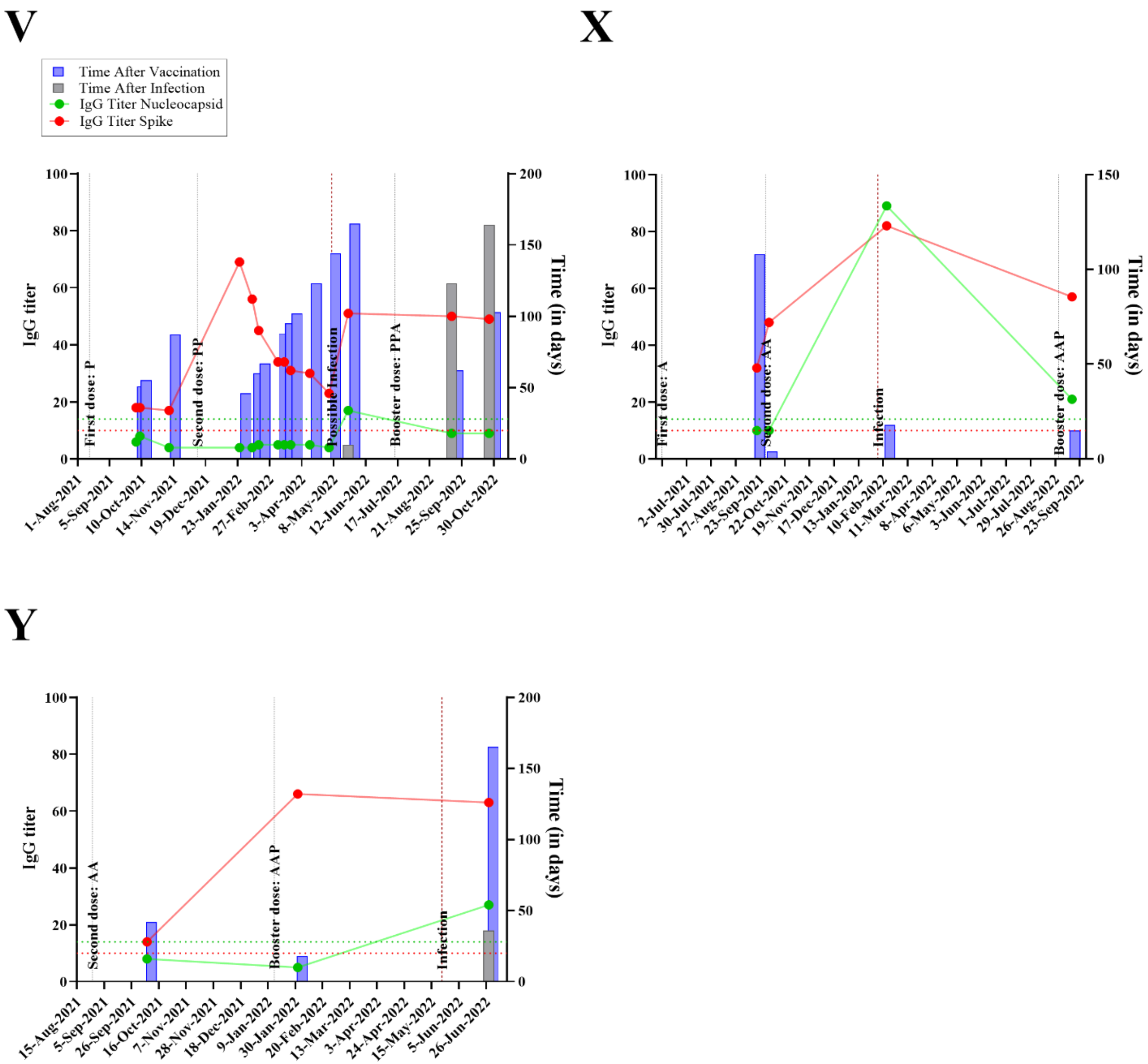
V
X
Y
Time After Vaccination
Time After Infection
IgG Titer Nucleocapsid
IgG Titer Spike
IgG titer
Time (in days)
First dose: P
Second dose: PP
Possible Infection
Booster dose: PPA
First dose: A
Second dose: AA
Infection
Booster dose: AAP
Second dose: AA
Booster dose: AAP
Infection
1-Aug-2021
5-Sep-2021
10-Oct-2021
14-Nov-2021
19-Dec-2021
23-Jan-2022
27-Feb-2022
3-Apr-2022
8-May-2022
12-Jun-2022
17-Jul-2022
21-Aug-2022
25-Sep-2022
30-Oct-2022
2-Jul-2021
30-Jul-2021
27-Aug-2021
23-Sep-2021
22-Oct-2021
19-Nov-2021
17-Dec-2021
13-Jan-2022
10-Feb-2022
11-Mar-2022
8-Apr-2022
6-May-2022
3-Jun-2022
1-Jul-2022
29-Jul-2022
26-Aug-2022
23-Sep-2022
15-Aug-2021
5-Sep-2021
26-Sep-2021
16-Oct-2021
7-Nov-2021
28-Nov-2021
18-Dec-2021
9-Jan-2022
30-Jan-2022
20-Feb-2022
13-Mar-2022
3-Apr-2022
24-Apr-2022
15-May-2022
5-Jun-2022
26-Jun-2022

Figure S1: Time course of IgG titers and relevant events in each individual. The x-axis indicates the temporal analysis of each individual, infection and vaccination events are indicated by vertical lines. Each vaccine dose and vaccination scheme are shown according to the initial letter of the vaccine's name (A, for AstraZeneca, C for CoronaVac, J for Janssen, P for Pfizer). The anti-N (green) and anti-S (red) IgG titers are reported on the left y-axis, with dashed lines indicating the positivity cutoff. The right y-axis corresponds to time in days after infection (gray) or administration of the last vaccine dose (blue), indicated by the vertical bars. Asymptomatic cases are indicated as “Possible Infections”. “Infection”, indicates individuals who declared a positive RT-qPCR or antigen SARS-CoV-2 test.

Figure S2 A-B

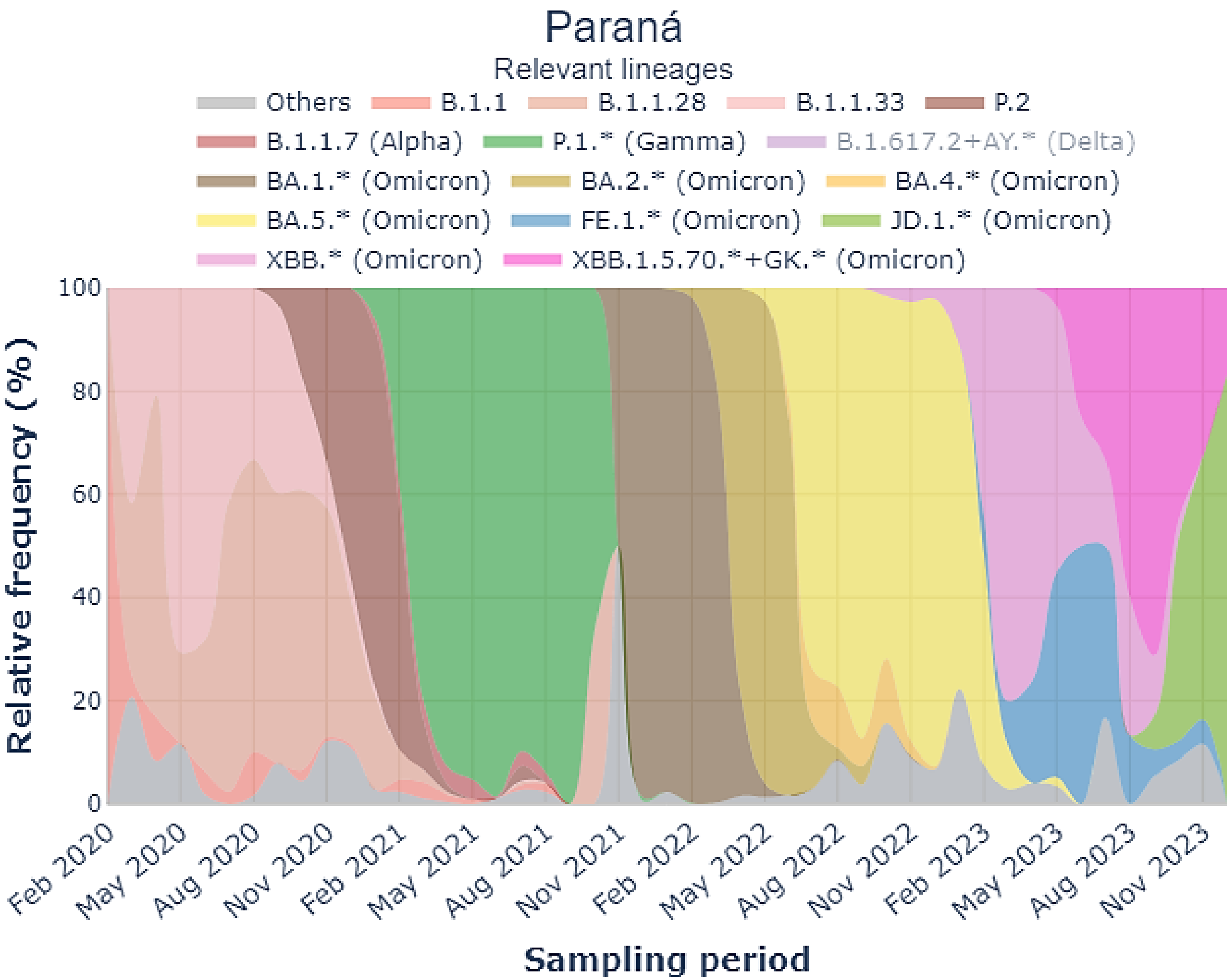

Figure S2 A: Relative frequency of SARS-CoV-2 variants circulating in the Paraná state in Brazil from 2020 to 2023. **Source:** https://www.genomahcov.fiocruz.br/dashboard-pt/

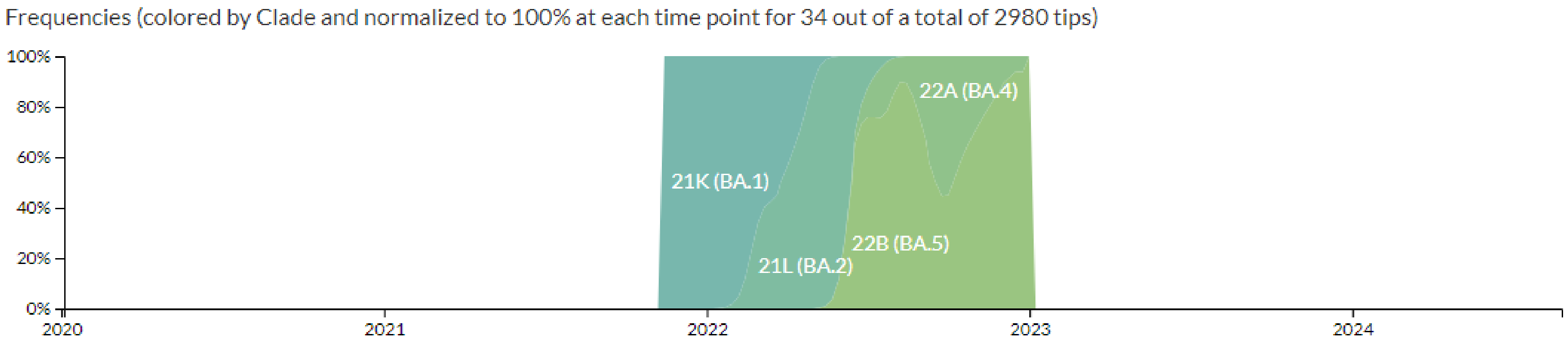

Figure S2 B: Frequency of SARS-CoV-2 variants circulating in South America during the time of this study. **Source:** https://nextstrain.org/ncov/gisaid/south-america/

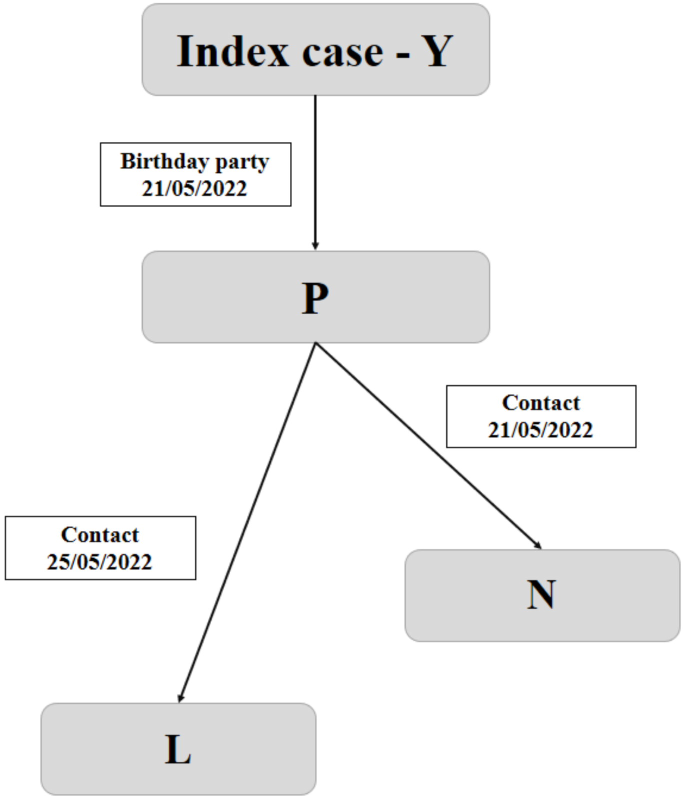

Figure S3: Descriptive flowchart of the clinical course of COVID-19 infection in cases related to individual N, representing the individuals' contact history. Description of cases: On 23/05/2022 the Index case (individual Y) test positive for SARS-CoV-2 (antigen test). Individuals code N and P had no negative symptoms and tested negative for SARS-CoV-2 antigen. On 25/05/2022 individual N has no symphotms and individual P reported fever and chills, sore throat and strong cough. On 26/05/2022 individual N and P tested positive for SARS-CoV-2 antigen; P reported sore throat, strong cough, congestion and running nose, while individual N reported a light sore throat. On 27/05/2022, individual N tested positive for SARS-CoV-2 based on RT-qPCR. This sample was subjected to SARS-CoV-2 genome sequencing. At this stage individual N reported fever and chills, while individual P had a sore throat, cough, congestion with running nose and loss of smell. On 28/05/2022 individual P again reported similar symptoms with cough, running nose and loss of smell. On this date, individual N, experienced a strong cough, with fever and chills. At this stage, individual L tested positive for SARS-CoV-2 antigen and reported fever and chills, with sore throat and a cough.. On 29/05/2022 individual P only reported loss of smell, individual N, reported cough and sore throat and individual L slightly recovered. On 30/05/2022 individuals P and L were fully recovered, and individual N reported cough, sore throat, loss of smell. On 31/05/2022 individual N fully recovered.
